# Dimerisation of Dipiperidinoacetylene: Convenient Access to Tetraamino‐1,3‐Cyclobutadiene and Tetraamino‐1,2‐Cyclobutadiene Metal Complexes

**DOI:** 10.1002/chem.201904726

**Published:** 2019-11-19

**Authors:** Ludwig Hackl, Alex R. Petrov, Thomas Bannenberg, Matthias Freytag, Peter G. Jones, Matthias Tamm

**Affiliations:** ^1^ Institut für Anorganische und Analytische Chemie Technische Universität Braunschweig Hagenring 30 38106 Braunschweig Germany

**Keywords:** carbenoids, cyclic bent allenes, cyclobutadienes, diaminoacetylenes, four-membered rings

## Abstract

The reaction of 1,2‐dipiperidinoacetylene (**1**) with 0.5 equivalents of SnCl_2_ or GeCl_2_⋅dioxane afforded the 1,2,3,4‐tetrapiperidino‐1,3‐cyclobutadiene tin and germanium dichloride complexes **2 a** and **2 b**, respectively. A competing redox reaction was observed with excess amounts of SnCl_2_, which produced a tetrapiperidinocyclobutadiene dication with two trichlorostannate(II) counterions. Heating neat **1** to 110 °C for 16 h cleanly produced the dimer 1,3,4,4‐tetrapiperidino‐3‐buten‐1‐yne (**3**); its reaction with stoichiometric amounts of SnCl_2_ or GeCl_2_⋅dioxane furnished the 1,3,4,4‐tetrapiperidino‐1,2‐cyclobutadiene tin and germanium dichloride complexes **4 a** and **4 b**, respectively. Transition‐metal complexes containing this novel four‐membered cyclic bent allene (CBA) ligand were prepared by reaction of **3** with [(tht)AuCl], [RhCl(CO)_2_]_2_, and [(Me_3_N)W(CO)_5_] to form [(CBA)AuCl] (**5**), [(CBA)RhCl(CO)_2_] (**6**), and [(CBA)W(CO)_5_] (**7**). The molecular structures of all compounds **2**–**7** were determined by X‐ray diffraction analyses, and density functional theory (DFT) calculations were carried out to rationalise the formation of **3** and **4 a**.

## Graphical abstract

**Cyclic bent allenes as ligands**: The thermal dimerisation of 1,2‐dipiperidinoacetylene affords 1,3,4,4‐tetrapiperidino‐3‐buten‐1‐yne, which provides access to main‐group and transition‐metal complexes of a strongly electron‐donating four‐membered cyclic bent allene ligand. The formation of these complexes as well as the dimerisation of 1,2‐dipiperidinoacetylene were also studied by computational methods.

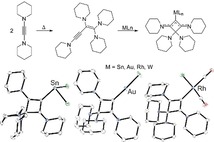

## Introduction

**Figure 1:**
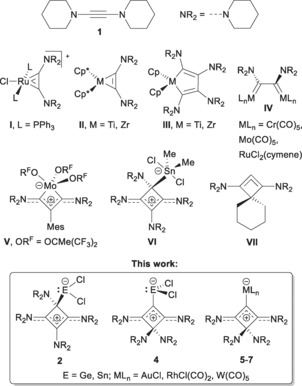
Selected complexes of dipiperidinoacetylene (**1**); Mes=2,4,6‐trimethylphenyl.

Diaminoacetylenes, or ynediamines, have been known since 1964, when Viehe and Reinstein reported the synthesis of 1,2‐bis(diethylamino)acetylene from 1,1‐dichloro‐2‐fluoroethylene and lithium diethylamide.^1^ The rather laborious nature of this and related protocols has prevented broad application in organic synthesis,^2^ whereas an interesting reactivity towards organotransition‐metal complexes was discovered.^3^ In 2010, our group established a novel synthetic approach that relies on a Fritsch–Buttenberg–Wiechell rearrangement upon lithiation of 2,2‐dibromo‐1,1‐ethylenediamines and provides convenient access to diaminoacetylenes such as 1,2‐dipiperidinoacetylene (**1**).^4^ With **1** and related species in hand, several mono‐ and bimetallic transition‐metal complexes have been prepared, such as ruthenium(II) complex **I**, in which the long C−C bond indicates that the alkyne acts as a chelating four‐electron diaminodicarbene ligand (Figure 1).^5^ Significantly shorter C−C bonds were found in the decamethylmetallocene (Cp*_2_ m, Cp*=C_5_Me_5_, M=Ti, Zr) complexes **II**, which are therefore best described as metallacyclopropene species. In contrast, the sterically less demanding titanocene (Cp_2_Ti, Cp= C_5_H_5_) and zirconocene (Cp_2_Zr) complex fragments accommodate two alkyne ligands and form the metallacyclopentadienes **III**.^6^ The diaminodicarbene character of **1** was also discovered by isolation of the homobimetallic complexes **IV**, whereas heterobimetallic Ru–Pd complexes were obtained by treatment of **I** with [(CH_3_CN)_2_PdCl_2_].^5^ Compound **1** also reacted as a vicinal dicarbenoid towards main‐group Lewis acids such as BPhCl_2_, with formation of a diborane adduct;^7^ however, B−C bond activation and 1,2‐carboboration was found when BPh_3_ or cyclic boroles such as 1‐mesityl‐2,3,4,5‐tetraphenylborole were used.^7^, ^8^ Similar reactivity was found for diborane and dialane species, which proceeded with diboration and dialumination of the C≡C triple bond in **1**.^9^

Unprecedented reactivity was also observed upon reaction of **1** with the molybdenum alkylidyne complex [MesC≡Mo{OCMe(CF_3_)_2_}_3_] (Mes=2,4,6‐trimethylphenyl), which afforded the paramagnetic metallacyclobutadiene (MCBD) complex **V** with a triplet ground state (*S=*1) through cleavage of the carbon–carbon triple bond and insertion of the alkylidyne moiety.^10^ In contrast to conventional MCBD species,^11^ this complex is best described as a Mo^IV^ complex containing an anionic diaminodicarbene ligand with strong π‐conjugation and electron delocalisation within the backbone NC_3_N unit (Figure 1). In principle, the same structural motif is present in the tin(IV) complex **VI**, which was obtained by Braunschweig et al. from the reaction of **1** with dimethyltin dichloride. The stannane adduct **VI** was described as a tin‐stabilised cyclobutadienyl system formed by [2+2] cycloaddition in the presence of the Lewis acid Me_2_SnCl_2_,^12^ which resembles the reactivity described much earlier by Viehe for the cyclisation of bis(dimethylamino)acetylene upon protonation or alkylation with triethylammonium bromide or methyl iodide, respectively.13a Furthermore, reaction of the former, the hydrobromide species, with elemental bromine gave the tetrakis(dimethylamino)cyclobutenediylium dibromide [C_4_(NMe_2_)]Br_2,_ which contains a rare example of an authenticated, albeit not structurally characterised “cyclobutadiene dication”.^14^ Independently of the work reported by the Braunschweig group on the isolation of the tin(IV) adduct **VI**,^12^ we have studied the reaction of dipiperidinoacetylene (**1**) towards germanium(II) and tin(II) chloride, the original goal being to produce 1:1 complexes for subsequent preparation of heavier Group 14 diaminocyclopropenylidene species of the type [(R_2_NC)_2_E] (E=Ge, Sn).^15^ Instead, as we wish to report herein, the zwitterionic 2:1 adducts **2** were isolated in a manner analogous to the formation of **VI** (Figure 1). By serendipity, however, the formation of the isomeric forms **4** was also observed, which could be ascribed to the presence of trace amounts of 1,1,2,4‐tetrapiperidino‐1‐buten‐3‐yne (**3**).

Compounds **4 a,b** can be viewed as complexes of a tetraamino‐1,2‐cyclobutadiene that is closely related to the all‐carbon four‐membered‐ring allene **VII** described by Bertrand.^16^ Compound **VII** was generated from a protonated precursor by reaction with lithium diisopropylamide (LDA) and shown to be persistent at low temperature in solution, presumably with stabilisation through lithium coordination; this was exploited for the preparation of the transition‐metal complexes [(**VII**)MCl(COD)] and [(**VII**)MCl(CO)_2_] (M=Rh, Ir, COD=1,5‐cyclooctadiene).^16^ We wish to show in this contribution that clean dimerisation of **1** to the corresponding 1‐buten‐3‐yne **3** can be achieved at elevated temperature and that **3** serves as a suitable starting material for the preparation of main‐group element and also transition‐metal complexes such as **4**–**7**. The ligand properties of this novel four‐membered carbenoid will be assessed and compared to other acyclic and cyclic bent allene (CBA) systems,^17^, ^18^, ^19^, ^20^ which have emerged as powerful ancillary ligands in organotransition‐metal chemistry and homogeneous catalysis.^21^

## Results and Discussion

### SnCl_2_‐ and GeCl_2_‐stabilised 1,3‐cyclobutadienes

**Scheme 1:**
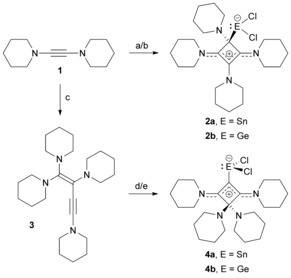
Preparation of SnCl_2_‐ and GeCl_2_‐stabilised cyclobutadienes: conditions and reagents: a) 0.5 equiv. SnCl_2_, MeCN, rt, 2.5 h; b) 0.5 equiv. GeCl_2_⋅dioxane, MeCN, rt, 2.5 h; c) 110 °C, 16 h; d) 1 equiv. SnCl_2_, THF, rt, 1.5 h; e) 1 equiv. GeCl_2_⋅dioxane, THF, rt, 1.5 h.

The reactions of dipiperidinoacetylene (**1**) with 0.5 equiv of SnCl_2_ or GeCl_2_⋅dioxane in acetonitrile at room temperature afforded the tin(II) and germanium(II) cyclobutadienyl complexes **2 a** and **2 b**, respectively, as orange, crystalline solids in moderate (56 % for **2 a**) to good yields (88 % for **2 b**) (Scheme 1). The ^13^C{^1^H} NMR spectra (in CDCl_3_) of **2 a**/**2 b** exhibit three signals each for the quaternary carbon atoms at 89.8/82.2 (C1), 112.6/114.0 (C2) and 173.7/172.9 ppm (C2+C4), which is in good agreement with the chemical shifts reported for the tin(IV) adduct **VI**, 78, 113, and 172 ppm (in C_6_D_6_),^12^ and confirms the formation of four‐membered rings with *C*_s_‐symmetry. The observation of the signal for the tin‐coordinated carbon atom in **2 a** at lower field (Δ*δ*=12 ppm) compared with **VI** is in agreement with the trend observed for other systems, for example N‐heterocyclic carbene (NHC) tin(II) and tin(IV) complexes.^22^ For **2 a**, ^119^Sn NMR spectroscopy revealed a signal at 161.9 ppm, which is in good agreement with the chemical shift (113.3 ppm) reported for an SnCl_2_ complex containing the carbon‐bound ylidic Wittig ligand Ph_3_P=CMe_2_.^23^

**Figure 2:**
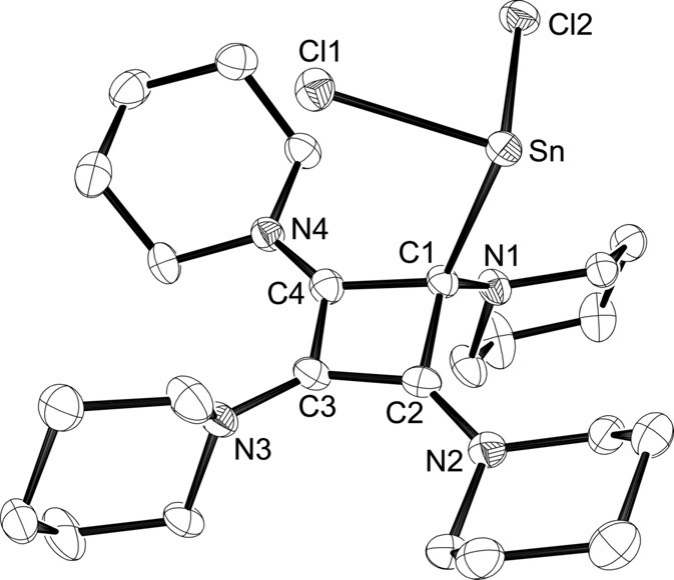
ORTEP diagram of **2 a** with thermal displacement parameters drawn at the 50 % probability level. Hydrogen atoms and a disordered position of the piperidyl ring at C3 have been omitted for clarity. For selected bond lengths and angles, see Table 1.

Crystals suitable for X‐ray diffraction analysis were obtained by diffusion of *n*‐pentane into a saturated solution in dichloromethane (**2 a**) or cooling a saturated solution in acetonitrile (**2 b**); **2 a** and **2 b** are isotypic and crystallise in the space group *P*2_1_/*c*. The molecular structure of **2 a** is shown in Figure 2, whereas that of **2 b** can be found in the Supporting Information (Figure S1). Both four‐membered rings are planar to within a mean deviation of 0.02 Å. Pertinent structural parameters are given in Table 1. As expected, the Sn and Ge atoms reside in acute trigonal‐pyramidal environments with Cl‐Sn‐Cl and Cl‐Ge‐Cl angles of 93.84(3)° and 95.613(12)°, respectively. The Sn−C1 and Ge−C1 bond lengths are 2.320(3) and 2.1249(12) Å and fall in the range reported for the corresponding carbon–element bonds in complexes of the type (NHC)ECl_2_ (E=Sn, Ge).^22^ The complexes (Ph_3_P=CMe_2_)ECl_2_ feature similar bond lengths of 2.3518(14) (Sn−C) and 2.1535(19) Å (Ge−C),^23^ whereas shorter bonds were found the ECl_2_ adducts of the four‐membered cyclic diphosphete [HCP(NMe_2_)_2_]_2_, namely Sn−C=2.267(3), Ge−C=2.070(3) Å.^24^ The structural features within the C_4_N_4_ unit are virtually identical to those reported for the corresponding tin(IV) adduct **VI**,^12^ with short C−C and C−N bonds along the N2‐C2‐C3‐C4‐N4 chain indicating a high degree of π‐conjugation and electron delocalisation. Accordingly, the piperidine groups at N2 and N4 are in a coplanar arrangement with the four‐membered ring (e.g. two absolute torsion angles each of the types C_pip_‐N2‐C2‐C1/3 and C_pip_‐N4‐C4‐C1/3 for **2 a** are less than 8°), whereas roughly perpendicular orientations are found at N1 and N3.

**Table 1:** Selected bond lengths and angles for the complexes **2** and **4**.^[a]^

Bond length [Å]	**2 a**	**2 b**	**4 a**	**4 b**
E−C1	2.320(3)	2.1249(12)	2.2444(2)	2.039(3)
C1−C2	1.558(5)	1.570 (2)	1.414(2)	1.424(3)
C1−C4	1.521(4)	1.524(2)	1.421(2)	1.415(4)
C2−C3	1.402(4)	1.412(2)	1.555(2)	1.571(3)
C3−C4	1.417(5)	1.414(2)	1.568(2)	1.559(3)
C4−N4	1.318(4)	1.308(2)	1.320(2)	1.313(3)
C3−N3	1.402(5)	1.406(2)	1.458(2)	1.450(3)
Bond angle [°]				
Cl1‐E‐Cl2	93.84(3)	95.613(12)	93.310(17)	97.18(3)
C1‐E‐Cl1	93.08(8)	95.30(3)	94.19(5)	101.57(7)
C1‐E‐Cl2	96.88(9)	98.70(3)	94.62(5)	93.33(7)
C2‐C1‐C4	81.3(2)	81.51(9)	89.86(19)	89.9(2)

[a] For comparison with calculated geometric parameters, see Table S4 in the Supporting Information.

It should be noted that attempts to improve the yield of **2 a** by variation of the stoichiometry and reaction time furnished grey metallic precipitates, in particular when an excess of tin(II) chloride and long reaction times (about 60 h) were employed. This indicates the formation of elemental tin through a concurrent redox process. Workup afforded a yellow solid and single crystals were obtained by diffusion of *n*‐hexane into a saturated THF solution. X‐ray diffraction analysis revealed the formation of the tetrapiperidinocyclobutadiene dication with two trichlorostannate(II) counterions (compound **S1**, Figures S7 and S8, Supporting Information). To the best of our knowledge, this compound represents the first structurally characterised salt containing a cyclobutadiene dication;^13^ however, its isolation in pure form was hampered by the formation of a mixture of salts containing extended chloride‐bridged polystannate anions of the type [Sn_*n*_Cl_2*n*+1_]^−^.^25^ When an excess of GeCl_2_ was used, another side product **S2** was isolated and structurally characterized (Figures S9 and S10), revealing *trans*‐addition of two GeCl_3_ units across the C−C triple bond in **1**. These findings show that the stoichiometry and reaction conditions need to be carefully balanced to obtain optimum yields of **2 a** and **2 b** and to avoid a competing one‐ or two‐electron oxidation of the electron‐rich diaminoacetylene **1**.

### SnCl_2_‐ and GeCl_2_‐stabilised 1,2‐cyclobutadienes

**Figure 3:**
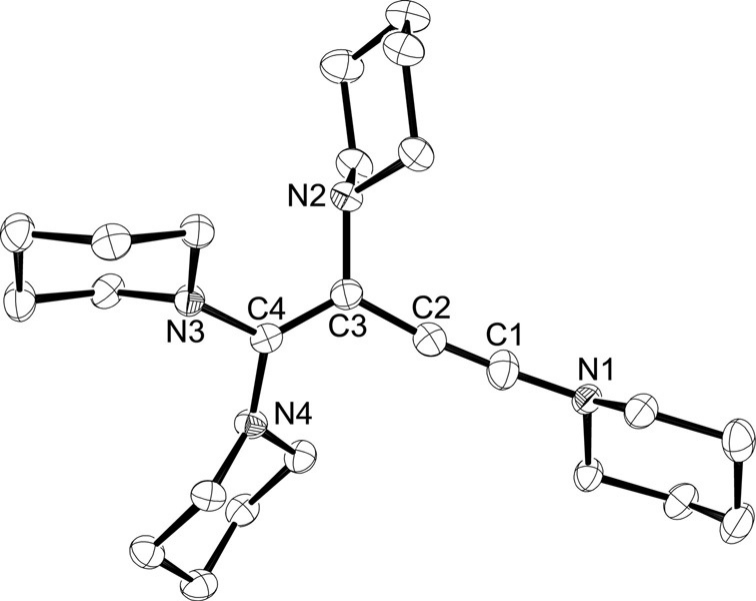
ORTEP diagram of **3** with thermal displacement parameters drawn at the 50 % probability level. Hydrogen atoms and a disordered position of the piperidyl ring and C1 have been omitted for clarity. Selected bond lengths [Å] and angles [°]: C1−C2 1.193(2), C2−C3 1.431(2), C3−C4 1.369(2), C3−N2 1.4486(14), C4−N3 1.3949(13), C4−N4 1.3944(14); C2‐C1‐N1 176.6(3), C2‐C3‐C4 123.08(10), N4‐C4‐N3 112.69(9).

Another side reaction was also observed by serendipity: when diaminoacetylene **1**, usually stored at −40 °C under argon atmosphere, was left at room temperature for several days, a significant increase in viscosity of the liquid was observed. ^1^H NMR spectroscopy revealed the formation of a new, less symmetric species. Subsequently, we tested different reaction conditions to isolate the new compound in pure form; heating **1** to 110 °C without any solvent for 16 h proved to be the best method. An orange‐brownish resin was obtained and suspended by stirring in hexamethyldisiloxane to afford 1,1,2,4‐tetrapiperidino‐1‐buten‐3‐yne (**3**) as a beige powder after filtration in high yield (92 %, Scheme 1). Further investigations, including 2D‐NMR spectroscopy and mass spectrometry, confirmed the formation of enyne **3** as the dimerisation product of **1**. For instance, the carbon atoms along the C=C−C≡C chain give rise to four ^13^C NMR signals at 157.8, 102.6, 59.9, and 101.8 ppm, reflecting the asymmetric charge distribution within the C_4_ chain. In addition, the molecular structure was determined unequivocally by X‐ray diffraction analysis of single crystals obtained from cooling a saturated THF solution to −40 °C (Figure 3). Given that the piperidine ring at N1 is disordered over two positions, the structural parameters must be interpreted with caution. Nevertheless, the expected connectivity is confirmed; the molecule is slightly twisted, with the strongest deviation observed for the N3‐C4‐N4 unit, which subtends an interplanar angle of 25.6° with the enyne plane containing the carbon atoms C1–C4.

The formation of **3** resembles the dimerisation of acetylene to vinylacetylene (3‐buten‐1‐yne), which is usually performed in the presence of copper (Nieuwland) catalysts.^26^ Vinylacetylene represents the most stable isomer on the C_4_H_4_ potential‐energy surface (PES), and its formation from acetylene was calculated to be exothermic with Δ*H*°=−40.3 kcal mol^−1^ at the CCSD(T) level of theory.^27^ It has been proposed that the dimerisation of acetylene proceeds with formation of a 1,4‐biradical followed by a [1,3]‐hydrogen shift. We were able to find a plausible mechanism for the formation of **3** that builds on the “hidden dicarbene nature”^28^ of diaminoacetylenes and involves C−C coupling between two molecules of **1**, affording a 1,4‐dicarbene intermediate, and a subsequent 1,3‐migration of one piperidyl group (Figure S35, Supporting Information). Our density functional theory (DFT) calculations at the B97‐D/6–311G(d,p) level of theory reveal a barrier of 15.2 kcal mol^−1^ (Δ*G*° referred to two equivalents of **1**) for the rate‐determining C−C coupling step, and the formation of **3** being exergonic with Δ*G*°=−36.3 kcal mol^−1^ (Δ*H*°=−49.9 kcal mol^−1^).

The reactions of enyne **3** with one equiv of SnCl_2_ or GeCl_2_⋅dioxane in THF at room temperature afforded yellowish‐white precipitates, and the dichlorostannylene and dichlorogermylene complexes **4 a** and **4 b**, respectively, were isolated in good yield (approx. 77 %) by filtration, extraction with dichloromethane, and evaporation (Scheme 1). NMR spectroscopic characterisation indicated the formation of *C*_2*v*_‐symmetric compounds with two different types of piperidine units. Notably, the ^13^C NMR spectra (in CDCl_3_) of **4 a**/**4 b** show three signals each at 180.1/179.2 (C2+C4), 148.1/137.2 (C1), and 97.7/95.9 ppm (C3) for the ring carbon atoms. The two lowfield signals can be assigned to the central NC_3_N moiety, which is in excellent agreement with the chemical shifts of 185.7 and 151.6 ppm reported for the cyclic allene **VII**, or rather its lithium adduct.^16^ Similar ranges are also found for transition‐metal complexes of **VII** (see below). Strong π‐conjugation across the diaminoallene NC_3_N unit and consequent hindered rotation around the exocyclic C−N bonds gives rise to broad ^1^H NMR signals for the 2,6‐CH_2_ hydrogen atoms of the two flanking piperidyl substituents.

**Figure 4:**
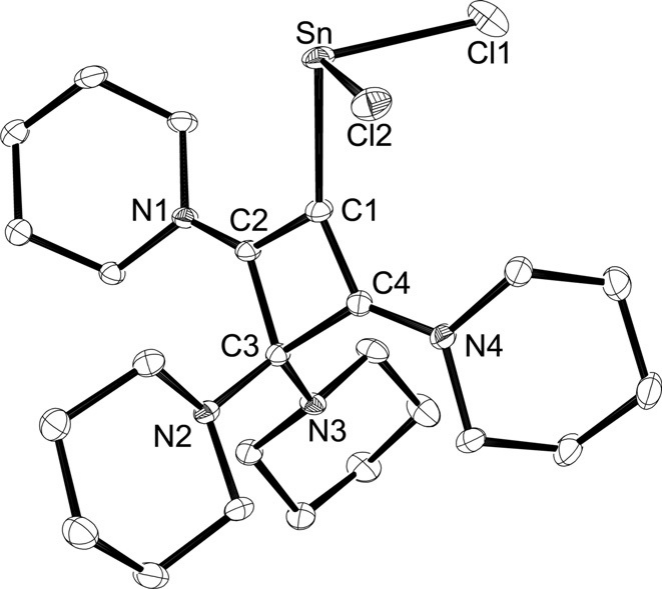
ORTEP diagram of **4 a** in **4 a**⋅CHCl_3_ with thermal displacement parameters drawn at the 50 % probability level. Hydrogen atoms and the molecule of chloroform have been omitted for clarity. For selected bond lengths and angles, see Table 1.

Single crystals of **4 a**⋅CHCl_3_ and **4 b** were subjected to X‐ray diffraction analysis; the molecular structure of the tin compound is presented in Figure 4, whereas that of the germanium derivative is shown in the Supporting Information (Figure S4). The four‐membered rings are planar to within 0.03 Å. Pertinent structural data are given in Table 1. With lengths of 2.2444(2) and 2.039(3) Å, the Sn−C1 and Ge−C1 bonds are shorter than those of N‐heterocyclic carbene adducts of the type (NHC)SnCl_2_ and (NHC)GeCl_2_,^22^ and are also clearly less than the values reported for similar complexes containing abnormal NHC, cyclic alkyl(amino) carbene (CAAC), and carbodiphosphorane ligands.^29^ The carbon–carbon and carbon–nitrogen bond lengths within the N1‐C2‐C1‐C4‐N4 unit lie between the expected values for single and double bonds, indicating a high degree of π‐conjugation and electron delocalisation. The tin and germanium atoms reside in acute trigonal‐pyramidal environments with Cl‐Sn‐Cl and Cl‐Ge‐Cl angles of 93.310(17)° and 97.18(3)°, respectively; these units are oriented in a staggered fashion towards the allene moieties in **4 a** and **4 b**. However, absolute N1‐C2‐C1‐E and N4‐C4‐C1‐E torsion angles of approximately 30° reveal twisted arrangements, in which the Ge and Sn atoms are displaced by 0.62 and 0.70 Å respectively from the C2‐C1‐C4 place.

**Figure 5:**
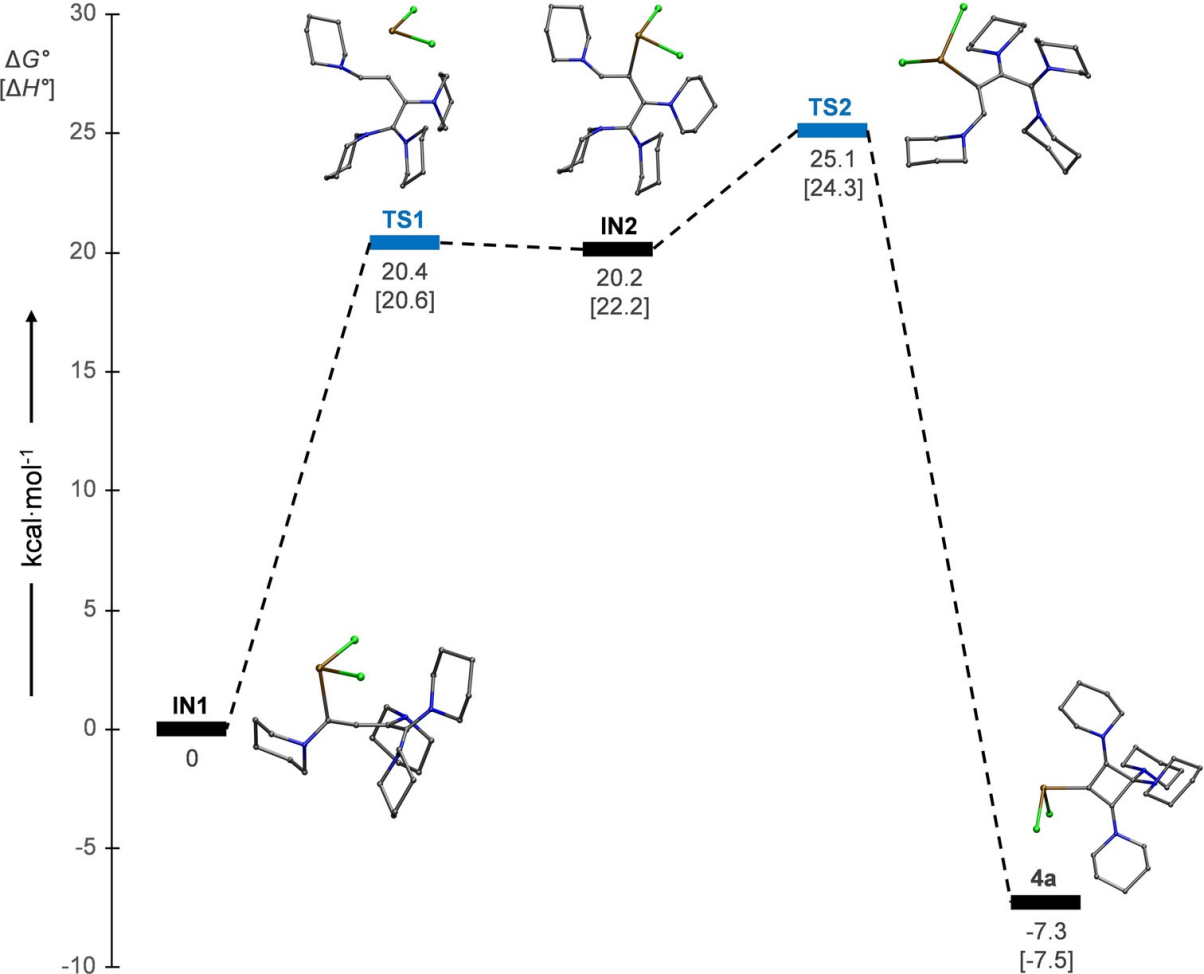
Calculated energy profile for the formation of **4 a**, scaled to standard Gibbs free energies (Δ*G*°); standard enthalpies (Δ*H*°) are given in square brackets.

The formation of **4 a** from **3** was also studied by DFT calculations at the B97‐D/6–311G(d,p) level of theory. The first step involves the strongly exergonic formation of intermediate **IN1**, which is formed by addition of SnCl_2_ at the terminal acetylenic carbon atom C1 (Δ*G*°=−34.8 kcal mol^−1^). It should be noted, however, that the large magnitude of this energy is not meaningful, given that the calculation involved the hypothetic high‐energy gas‐phase species SnCl_2_. Therefore, the energy profile shown in Figure 5 is referenced to **IN1**, which further undergoes a 1,2‐shift of the SnCl_2_ moiety to carbon atom C2 to give **IN2**, followed by ring closure and C−C bond formation between the terminal carbon atoms C1 and C4. The overall reaction is exergonic by Δ*G*°=−7.3 kcal mol^−1^ and involves a maximum barrier of Δ*G*°=25.1 kcal mol^−1^. Nevertheless, the 1,2‐cyclobutadiene complex **4 a** does not represent the global minimum on the energy hypersurface, and the corresponding 1,3‐cyclobutadiene adduct **2 a** is stabilised by Δ*G*°=−5.9 kcal mol^−1^ (Figure S36, Supporting Information). Interconversion between **2 a** and **4 a** cannot be observed experimentally, and the formation of isomer **2 a** from two equivalents of **1** and SnCl_2_ can be rationalised by a similar mechanism as proposed for the tin(IV) adduct **VI**.^12^

### Transition‐metal 1,2‐cyclobutadiene complexes

**Scheme 2:**
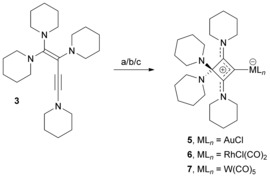
Synthesis of transition‐metal 1,2‐cyclobutadiene complexes; conditions and reagents: a) 1 equiv. [(tht)AuCl], THF, rt, 2 h, 82 %; b) 0.5 equiv. [RhCl(CO)_2_]_2_, toluene, rt, 4 h, 84 %; c) 1 equiv. [(NMe_3_)W(CO)_5_], THF, 50 °C, 16 h, 36 %.

**Figure 6:**
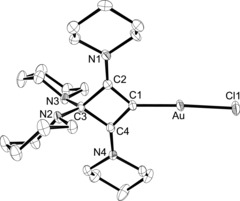
ORTEP diagram of **5** in **5**⋅CH_2_Cl_2_ with thermal displacement parameters drawn at the 50 % probability level. Hydrogen atoms and the molecule of dichloromethane have been omitted for clarity. For selected bond lengths and angles, see Table 2.

The ability of enyne **3** to form cyclic bent allene (CBA) complexes was further explored by reaction with various transition‐metal precursors (Scheme 2). Treatment with [(tht)AuCl] (tht=tetrahydrothiophene) provided the gold(I) complex **5** as a white solid in good yield (82 %, Scheme 2). Ring closure and formation of a CBA ligand was confirmed by ^1^H and ^13^C{^1^H} NMR spectroscopy with three signals at 95.9 (C3), 123.6 (C1), and 179.2 (C2/C4) ppm for the ring carbon atoms. Diffusion of *n*‐hexane into a solution of **5** in CH_2_Cl_2_ gave single crystals of **5**⋅CH_2_Cl_2_ suitable for X‐ray diffraction analysis. The molecular structure (Figure 6) confirms the formation of a linear gold complex with a C1‐Au‐Cl angle of 176.77(11)°. At 2.001(4) Å, the Au−C1 bond length is similar to those established for NHC gold(I) complexes, compare 1.998(5) Å in [(IMes)AuCl] (IMes=1,3‐bis(2,4,6‐trimethylphenyl)imidazolin‐2‐ylidene).^30^ The four‐membered ring and the gold atom are coplanar to within 0.002 Å. To assess the steric properties of the CBA ligand in **5**, the buried volume descriptor (%*V*_Bur_) was determined as 30.5 % using the software SambVca 2 (see the Supporting Information for details).^31^ Comparison with the values calculated by Nolan and co‐worker^32^ for different NHC ligands showed that this value is larger than for a cyclohexylsubstituted NHC ligand 1,3‐bis(cyclohexyl)imidazolin‐2‐ylidene) (ICy, %*V*_Bur_=27.4 %), but smaller compared with the widely used aryl‐substituted NHCs IMes (%*V*_Bur_=36.5 %) and 1,3‐bis(2,6‐diisopropylphenyl)imidazolin‐2‐ylidene (IDipp, %*V*_Bur_=44.5 %).

To assess the donor strength of the novel CBA ligand by IR spectroscopy,^33^, ^34^, ^35^, ^36^ the rhodium(I) complex **6** was prepared by reaction of enyne **3** with 0.5 equiv. of [Rh(μ‐Cl)(CO)_2_]_2_ in toluene at room temperature. After evaporation of the solvent and washing with *n*‐hexane and diethyl ether, **6** was isolated as an orange‐brown solid in 84 % yield. The ^13^C{^1^H} NMR spectrum exhibits three doublets for the metal‐bound carbon atoms at 131.2 (C1, ^1^ *J*_C−Rh_=32 Hz), 186.9 (*trans*‐CO, ^1^ *J*_C−Rh_=52 Hz), and 184.9 ppm (*cis*‐CO, ^1^ *J*_C−Rh_=79 Hz). These chemical shifts are in excellent agreement with the data reported for the corresponding complex [(**VII**)RhCl(CO)_2_]. Similarly, the CO stretching frequencies reported for this complex ($\tilde{\nu }$ =1976, 2055 cm^−1^; $\tilde{\nu }$_av_=2016 cm^−1^) perfectly match those determined for **6** ($\tilde{\nu }$ =1978, 2056 cm^−1^; $\tilde{\nu }$_av_=2017 cm^−1^), confirming the strong electron‐donating ability of this type of 1,3‐diamino‐1,2‐cyclobutadiene ligands.^16^ Hence, these ligands appear to be stronger donor ligands than most cyclic and acyclic diaminocarbene ligands,^33^, ^34^, ^35^, ^36^ and similar or even lower values were only reported for a few other C‐donor ligands,^37^ such as five‐membered cyclic bent allenes,^17^ carbodicarbenes,^38^ carbodiphosphoranes,^39^ and N‐heterocyclic olefins.^18^, ^40^

**Figure 7:**
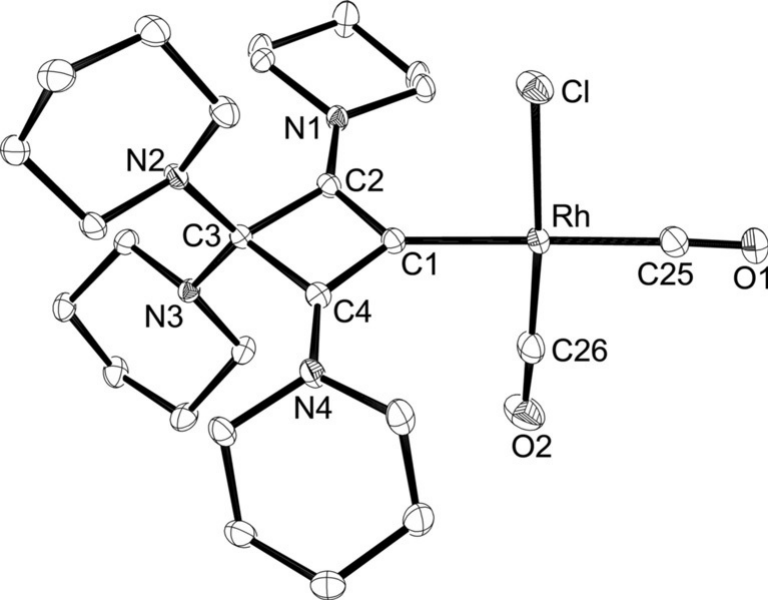
ORTEP diagram of **6** with thermal displacement parameters drawn at the 50 % probability level. The chloride and the *cis*‐carbonyl group show substitutional disorder. The hydrogen atoms and the minor disorder component have been omitted for clarity. For selected bond lengths and angles, see Table 2.

The molecular structure was additionally confirmed by X‐ray diffraction analysis (Figure 7). The structure is disordered, with exchange of the Cl and CO ligand sites, but is nevertheless reliable. The rhodium atom displays a square‐planar coordination sphere (mean deviation 0.008 Å), which subtends an interplanar angle of 66.6° with the CBA plane (C1‐C2‐C3‐C4; mean deviation 0.006 Å). The structural parameters within the CBA ligand closely resemble those in **5**, and the Rh−C1 bond length of 2.0602(14) Å is marginally greater than the 2.038(5) Å in [(**VII**)RhCl(CO)_2_].^16^

**Figure 8:**
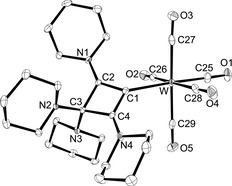
ORTEP diagram of **7** with thermal displacement parameters drawn at the 50 % probability level. Hydrogen atoms have been omitted for clarity. For selected bond lengths and angles, see Table 2.

Another transition‐metal carbonyl complex was prepared by reaction of enyne **3** with [(Me_3_N)W(CO)_5_] in THF. After stirring for 16 h at 50 °C the solvent was removed. The residue was washed with *n*‐hexane and recrystallised from diethyl ether/*n*‐hexane solution to provide the pentacarbonyltungsten(0) complex **7** as a yellow solid in relatively low yield (36 %). The molecular structure was established by X‐ray diffraction analysis (Figure 8), revealing a slightly distorted‐octahedral coordination geometry around the tungsten atom. Unlike the coplanar orientation in the gold(I) and rhodium(I) complexes **5** and **6**, the CBA ligand is tilted with regard to the metal atom as indicated by an W‐C1‐C3 angle of 158.40(16)° (Table 2), presumably because of steric interaction with the sterically more demanding W(CO)_5_ complex fragment; the tungsten atom lies 0.91 Å out of the plane of the four‐membered ring, which has a mean deviation of 0.05 Å, somewhat larger than in the other compounds presented here. The W−C1 bond length is 2.319(3) Å, which is slightly longer than usually found for [(NHC)W(CO)_5_] complexes, for example 2.282(3) Å in [(ICy)W(CO)_5_],^41^ 2.275(8) Å in [(^Me^IEt)W(CO)_5_] (^Me^IEt=1,3‐diethyl‐4,5‐dimethylimidazolin‐2‐ylidene), or 2.260(2) Å in [(IDipp)W(CO)_5_].^42^

**Table 2:** Selected bond lengths and angles for the transition‐metal complexes **5**–**7**.

Bond length [Å]	**5**	**6**	**7**
M−C1	2.001(4)	2.0602(14)	2.319(3)
C1−C2	1.404(5)	1.4064(19)	1.421(4)
C1−C4	1.413(5)	1.4139(19)	1.416(4)
C2−C3	1.558(5)	1.5537(18)	1.557(4)
C3−C4	1.564(5)	1.5522(19)	1.549(4)
C4−N4	1.314(5)	1.3265(18)	1.326(4)
C3−N3	1.452(4)	1.4585(17)	1.469(4)
Bond angle [°]			
C1‐M‐E_trans_^[a]^	172.01(12)	176.77(11)	178.77(6)
C1‐M‐C26	–	86.54(8)	85.29(11)
C2‐C1‐C4	90.5(3)	89.73(11)	88.0(2)
C1‐C2‐C3	95.2(3)	95.42(11)	95.8(2)
C2‐C3‐C4	79.7(3)	79.66(10)	78.8(2)
M‐C1‐C3	177.80(10)	178.31(12)	158.40(16)

[a] E_trans_=Cl (**5**); E_trans_=C25 (**6**,**7**).

The tungsten pentacarbonyl complex **7** was also investigated by IR spectroscopy. The IR spectrum shows bands for the four CO stretching modes $\tilde{\nu }$ (CO)=1866 (*A*_1_^(1)^), 1888 (*E*), 1942 (*B*_1_), 2047 (*A*_1_^(2)^) cm^−1^), as expected for a *C*_1_‐symmetric pentacarbonyl complex,^43^ although the *B*_1_ fundamental is only resolved as a shoulder. Comparison with reported values for phosphines (e.g. PPh_3_: $\tilde{\nu }$ (*A*_1_^(1)^)=1939; PMe_3_: $\tilde{\nu }$ (*A*_1_^(1)^)=1937 cm^−1^)^44^ and NHCs (e.g. 1,3‐diisopropylimidazolin‐2‐ylidene, IPr: $\tilde{\nu }$ (*A*_1_^(1)^)=1880 cm^−1^)^45^ further emphasises the strong donating ability of the CBA ligand.

## Conclusions

The reaction of dipiperidinoacetylene (**1**) with SnCl_2_ and GeCl_2_⋅dioxane afforded the expected 1,3‐cyclobutadiene tin and germanium dichloride complexes **2 a** and **2 b**. The clean conversion of **1** into its dimer tetrapiperidino‐3‐buten‐1‐yne (**3**), as discovered in this contribution, provided access to the 1,2‐cyclobutadiene isomers **4 a** and **4 b**. These complexes feature the tetrapiperidino‐1,2‐cyclobutadiene ligand as a new four‐membered addition to the family of cyclic bent allenes (CBA), and the cyclisation of **3** in the presence of Lewis acidic main‐group and transition‐metal complex fragments represents a general entry to this class of CBA complexes, as demonstrated by the successful preparation of the gold(I), rhodium(I) and tungsten(0) complexes **5**–**7**. The strong electron‐donating ability indicated by IR spectroscopy hints at the potential of this carbenoid ligand for applications in homogeneous catalysis, with the obvious possibilities of tuning this system by variation of the diaminoacetylene precursor or by post‐functionalisation at the terminal aminal group.

## Conflict of interest

The authors declare no conflict of interest.

## Supporting information

As a service to our authors and readers, this journal provides supporting information supplied by the authors. Such materials are peer reviewed and may be re‐organized for online delivery, but are not copy‐edited or typeset. Technical support issues arising from supporting information (other than missing files) should be addressed to the authors.

Supplementary

Supplementary
