# Supplementary material for: Dimerisation of Dipiperidinoacetylene: Convenient Access to Tetraamino‐1,3‐Cyclobutadiene and Tetraamino‐1,2‐Cyclobutadiene Metal Complexes
Source: Chemistry. 2019 Nov 19;25(70):16148–55. doi: 10.1002/chem.201904726 (PMC6973143; doi:10.1002/chem.201904726)
Supplement: Supplementary file 2 — Supplementary [file CHEM-25-16148-s002.pdf]

CHEMISTRY
A European Journal

Supporting Information

Dimerisation of Dipiperidinoacetylene: Convenient Access to Tetraamino-1,3-Cyclobutadiene and Tetraamino-1,2-Cyclobutadiene Metal Complexes

Ludwig Hackl, Alex R. Petrov, Thomas Bannenberg, Matthias Freytag, Peter G. Jones, and Matthias Tamm*[a]

chem_201904726_sm_miscellaneous_information.pdf
chem_201904726_sm_all_computed_structures.mol

Content

Synthetic details and procedures	3-7
X-Ray structure analyses	8-16
NMR-spectra	17-27
Buried Volume Calculations	28
Computational Details	29-34
References	35

Synthetic details and procedures

All reactions and manipulations were performed under an Argon atmosphere, using either standard Schlenk-line techniques or a Glovebox (MBraun). Solvents were purified by drying over Na/benzophenone (THF, *n*-hexane, diethylether) or CaH_2 (dichloromethane, acetonitrile, HMDSO) followed by distillation, and stored over Molecular Sieve (3 or 4 Å). **1**[1], $[(Me_3N)W(CO)_5]$[2] and [(tht)AuCl][3] were prepared according to literature procedures. $SnCl_2$, $GeCl_2$-dioxane complex and $[RhCl(CO)_2]_2$ were purchased from commercial sources (Sigma Aldrich, ABCR).

^{1}H-, ^{13}C{^{1}H} and ^{119}Sn-NMR spectra were recorded on Bruker AV 300, AVII300HD and AV-II 600. Chemical shifts (δ) are expressed in ppm and are given relative to the solvent residue signal ($CDCl_3$: δ_H = 7.26 ppm, δ_C = 77.16 ppm; C_6D_6: δ_H = 7.16 ppm, δ_C = 128.06 ppm) or external tetramethyltin (δ_{Sn} = 0 ppm). If no multiplicity is given for ^{13}C data the signal is a singulet. NMR assignments were made using additional 2D-NMR experiments.

Mass spectra were recorded on a MAT 4515 (Finnigan) using electron ionisation method.

Infrared spectra were measured on a Bruker Vertex 70 FTIR equipped with a Pike Technologies MIRacle attenuated total reflectance (ATR) with neat sample or a solution in dichloromethane.

Elemental analysis was performed by combustion and gas chromatographic analysis on an Elementar VarioMICRO Cube instrument.

Synthesis of 2a

Tin dichloride (143 mg, 0.75 mmol, 1 Eq.) was stirred in acetonitrile (5 mL) for 45 minutes, after which a white suspension had formed. Bis(piperidino)acetylene (300 mg, 1.55 mmol, 2.1 equiv), dissolved in acetonitrile (2 mL), was added quickly, whereupon the reaction mixture turned orange. After stirring for 2.5 h at ambient temperature the solvent was removed in vacuo. The orange residue was dissolved in THF (5 mL) and filtered over a short pad of Celite®. The solvent was removed in vacuo. **2a** was obtained as an orange solid (241 mg, 56%).

Crystals suitable for X-ray diffraction analysis were obtained by diffusion of *n*-pentane into a saturated solution of **2a** in dichloromethane.

^{1}H-NMR ($CDCl_3$, 300 MHz): δ = 3.88-3.58 (m, 8H, α-C*H*$_2$), 3.09-2.65 (m, 8H, α-C*H*$_2$), 1.99-1.76 (m, 4H, β-C*H*$_2$), 1.75-1.57 (m, 8H, β-γ-C*H*$_2$), 1.56-1.39 (m, 12H, β-γ-C*H*$_2$) ppm.

^{13}C{^{1}H}-NMR ($CDCl_3$, 74.5 MHz): δ = 173.7 (2C, Sn-C-(*C*≈N)$_2$), 112.6 (1C, *C*$_q$), 89.8 (1C, Sn-*C*), 54.0 (2C, α-*C*H$_2$), 51.2 (2C, α-*C*H$_2$), 49.4 (2C, α-*C*H$_2$), 27.4, 26.5, 26.4, 26.2, 25.0, 24.0, 23.8 (12C, β-γ-*C*H$_2$) ppm.

^{119}Sn-NMR ($CDCl_3$, 149.3 MHz): δ = 161.9 ppm.

EA (%): calculated for $C_{24}H_{40}N_4SnCl_2$: C: 50.20, H: 7.02, N: 9.76; found: C: 50.12, H: 7.046, N: 9.52.

Synthesis of 2b

To a stirred solution of germanium dichloride dioxane complex (110 mg, 0.48 mmol) in acetonitrile (4 mL), a solution of bis(piperidino)acetylene (232 mg, 1.21 mmol, 2.5 equiv.) in MeCN (1 mL) was added slowly at ambient temperature. After the addition of half of the reagent, a dark-red suspension formed; upon further addition the color changed to orange. The reaction mixture was stirred for 30 min. The clear yellow solution over a fluffy, brown precipitate was collected by filtration through a short pad of Celite®. The reaction mixture was further stored without stirring. Large orange crystals start to form upon storing the reaction mixture at ambient temperature; the crystallization was completed by storing the solution at +5°C. The crystalline solid was collected by decantation, washed twice with small amounts of cold acetonitrile and dried *in vacuo*. **2b** was obtained as an orange solid (253 mg, 88%).

Crystals suitable for X-ray analysis were obtained by cooling a concentrated solution of **2b** in acetonitrile to +5°C.

^{1}H-NMR ($CDCl_3$, 300 MHz): δ = 3.86–3.66 (m, 8H, α-C*H*$_2$), 2.86-.282 (m, 8H, α-C*H*$_2$), 1.96–1.78 (m, 4H, β-C*H*$_2$), 1.75–1.59 (m, 8H, β-γ-C*H*$_2$), 1.40–1.57 (m, 12H, β-γ-C*H*$_2$) ppm.

^{13}C{^{1}H}-NMR ($CDCl_3$, 74.5 MHz): δ= 172.9 (2C, Ge-C-(*C*≈N)$_2$), 114.0 (1C, *C*$_q$), 82.2 (1C, Ge-*C*), 53.7 (2C, α-*C*H$_2$), 51.7 (2C, α-*C*H$_2$), 49.4 (2C, α-*C*H$_2$), 27.3, 26.4, 26.2, 26.1, 25.5, 24.0, 23.8 (12C, β-γ-*C*H$_2$) ppm.

EA (%): calculated for $C_{24}H_{40}Cl_2GeN_4$: C 54.58, H 7.63, N 10.61; found: C 54.44, H 7.63, N 10.61.

Synthesis of 3

Neat bis(piperidino)acetylene (300 mg, 0.78 mmol) was stirred for 16 hours at 110°C. The yellow liquid turned orange during this time. The reaction mixture was cooled to room temperature, during which it solidified. To the sticky resin a small amount of HMDSO (~2 mL) was added and the mixture stirred for 30 min, during which the resin turned into a beige-orange powder. The supernatant was removed by cannula and the solid dried *in vacuo*. **3** was obtained as a beige solid (276 mg, 92%).

Crystals suitable for X-ray diffraction analysis were obtained by cooling a saturated solution of **3** in THF to -40 °C.

^{1}H-NMR (C_6D_6, 300 MHz): δ= 3.33-3.24 and 3.17-3.09 (2 x m, 2 x 4H, =C-(N- *α*-C*H*$_2$)$_2$), 3.04-2.98 (m, 4H, ≡-C-N-*α*-C*H*$_2$), 2.85-2.79 (m, 4H, =C-N-*α*-C*H*$_2$), 1.73-1.62 (m, 4H, =C-(N-*γ*-C*H*$_2$)$_2$), 1.58-1.37 (m, 18H, =C-N-*β*-CH$_2$, =C-(N-*β*-C*H*$_2$)$_2$, ≡-C-N-*β*-C*H*$_2$ bzw. -*γ*-C*H*$_2$), 1.26-1.15 (m, 2H, =C-N-*γ*-C*H*$_2$) ppm.

^{13}C{^{1}H}-NMR (C_6D_6, 74.5 MHz): δ= 157.8 (1C, N2-*C*=C), 102.6 (1C, ≡C-*C*=), 101.8 (1C, ≡*C*-N), 59.9 (1C, ≡*C*-C=), 55.8 (2C, ≡-C-N-*α*-*C*H$_2$), 51.4 and 51.5 (2C, =C-(N-*α*-*C*H$_2$)$_2$), 27.96 (1C, ≡C-N-*γ*-*C*H$_2$), 27.93 (1C, =-C-N-*γ*-*C*H$_2$), 27.87 (2C, =C-(N-*γ*-*C*H$_2$)$_2$), 26.4 and 26.5 (=C-(N-*β*-*C*H$_2$)$_2$), 26.1 (2C, =C-N-*β*-*C*H$_2$), 25.6 (2C, ≡-C-N-*β*-*C*H$_2$) ppm.

MS (EI; 70 eV) (m/z): M^+: 384.3, M-$C_5H_{10}N^+$: 300.3, $C_5H_{10}N^+$: 84.1.

EA (%): calculated for $C_{24}H_{40}N_4$: C: 74.95, H:10.48, N: 14.57; found: C: 74.76, H: 10.297, N:14.34.

Synthesis of 4a

Tin dichloride (26.9 mg, 0.142 mmol, 1.0 equiv) was suspended in THF (1 mL) and stirred for 30 min. To the reaction mixture **3** (60 mg, 0.156 mmol, 1.1 equiv) dissolved in THF (0.5 mL) was added slowly. The reaction mixture was stirred for 3 h at ambient temperature, during which a white suspension formed. The white-yellowish solid was filtered off, washed with THF (2 x 0.5 mL) and extracted with dichloromethane. The solvent was removed in vacuo and **4a** was obtained as a white solid (64 mg, 78%).

Crystals suitable for X-ray diffraction analysis were obtained by cooling a saturated solution of **5a** in $CHCl_3$ to -40 °C.

^{1}H-NMR ($CDCl_3$, 300 MHz): δ= 3.88-3.79 and 3.63-3.57 (2 x m, 2 x 4H, C2/C4-N-α-CH_2), 2.66-2.53 (br s, 8H, C3-(N-α-CH_2)$_2$), 1.80-1.73 (m, 4H, C2/C4-N-γ-CH_2), 1.72-1.58 (m, 8H, C2/C4- N-β-CH_2), 1.57-1.41 (m, 12H, C3-(N-β-γ-CH_2)$_2$) ppm.

^{13}C{^{1}H}-NMR ($CDCl_3$, 74.5 MHz): δ= 180.1 (2C, Sn-C-(*C*≈N)$_2$), 148.1 (1C, Sn-*C*), 97.7 (1C, *C3*), 52.5 (2C, C2/4-(N-α-*C*H$_2$)$_2$),), 50.5 (2C, C2/4-(N-α-*C*H$_2$)$_2$), 48.8 (4C, C3-(N-α-*C*H$_2$)$_2$), 26.7 (4C, C3-(N-β- *C*H$_2$)$_2$), 23.3, 25.0, 25.9, 26.3 (8C, β-γ-*C*H$_2$) ppm.

^{119}Sn-NMR ($CDCl_3$, 149.3 MHz): δ= 77.5 ppm.

EA (%): calculated for $C_{24}H_{40}N_4SnCl_2$: C: 50.20, H: 7.02, N: 9.76; found: C: 49.68, H: 7.085, N: 9.75.

Synthesis of 4b

Germanium dichloride dioxane complex (32.9 mg, 0.142 mmol, 1.0 equiv) was dissolved in THF (1 mL). To the reaction mixture **3** (60 mg, 0.156 mmol, 1.1 equiv) dissolved in THF (0.5 mL) was added slowly. The reaction mixture was stirred for 1.5 h at ambient temperature, during which a yellow suspension formed. The yellowish-white solid was filtered off, washed with THF (2 x 0.5 mL) and extracted with dichloromethane. The solvent was removed *in vacuo* and **4b** was obtained as a yellowish-white solid (56 mg, 76%).

Crystals suitable for X-ray diffraction analysis were obtained by diffusion of *n*-hexane into a solution of **4b** in CH_3Cl at -40 °C.

^{1}H-NMR ($CDCl_3$, 300 MHz): δ= 4.01-3.59 and 3.66-3.59 (2 x m, 2 x 4H, C2/C4-N-α-CH_2), 2.67-2.56 (br s, 8H, C3-(N-α-CH_2)$_2$), 1.86-1.75 (m, 4H, C2/C4-N-γ-CH_2), 1.73-1.61 (m, 8H, C2/C4- N-β-CH_2), 1.59-1.45 (m, 12H, C3-(N-β-γ-CH_2)$_2$) ppm.

^{13}C{^{1}H}-NMR ($CDCl_3$, 74.5 MHz): δ= 179.2 (2C, Ge-C-(*C*≈N)$_2$), 137.2 (1C, Ge-*C*), 95.9 (1C, *C3*), 50.9 (2C, C2/4-(N-α-*C*H$_2$)$_2$), 48.8 (4C, C3-(N-α-*C*H$_2$)$_2$), 47.8 (2C, C2/4-(N-α-*C*H$_2$)$_2$), 26.7 (4C, C3-(N-β- *C*H$_2$)$_2$), 23.4, 25.0, 26.0, 26.5 (8C, β-γ-*C*H$_2$) ppm.

EA (%): calculated for $C_{24}H_{40}N_4GeCl_2$: C: 54.58, H: 7.63, N: 10.61; found: C: 53.90, H: 7.632, N: 10.09.

Synthesis of 5

(tht)AuCl (41 mg, 0.13 mmol, 1 equiv) was added to a flask and THF (1 mL) added. **3** (50 mg, 0.13 mmol, 1 equiv) dissolved in THF (0.5 mL) was added slowly. The white-brown suspension was stirred for 2 h at ambient temperature. The white solid was isolated by filtration and washed with a little amount of cold THF (0.5 mL), dissolved in dichloromethane and the solvent was removed in vacuo, yielding **5** as a white solid.

The THF solution was reduced to half of the volume, and a second portion of product was then obtained by cooling to -40 °C followed by filtration. The total yield of the white gold complex **5** was 66 mg (82%).

Crystals suitable for X-ray diffraction analysis were obtained by diffusion of *n*-hexane into a solution of **5** in dichloromethane at -40 °C.

^{1}H-NMR ($CDCl_3$, 300 MHz): δ= 4.33-4.25 and 3.56-3.50 (2 x m, 2 x 4H, C2/C4-N-α-C*H*2), 2.65-2.46 (br s, 8H, C3-(N-α-C*H*2)2), 1.71-1.45 (m, 24H, β-γ-C*H*) ppm.

^{13}C{^{1}H}-NMR ($CDCl_3$, 75.1 MHz): δ= 179.2 (2C, Au-C-(*C*≈N)$_2$), 123.6 (1C, Au-*C*), 95.9 (1C, *C3*), 50.9 (2C, C2/4-(N-α-*C*H2)2), 48.9 (4C, C3-(N-α-*C*H2)2), 47.9 (2C, C2/4-(N-α-*C*H2)$_2$), 26.8 (4C, C3-(N-β- *C*H2)2), 26.6, 25.9, 25.2, 23.8 (8C, β-γ-*C*H_2) ppm.

EA(%): calculated for $C_{24}H_{40}N_4AuCl + CH_2Cl_2$: C: 42.78, H: 6.03, N: 7.98; found: C: 42.73, H: 5.825, N: 7.64.

Synthesis of 6

$[RhCl(CO)_2]_2$ (22.5 mg, 0.06 mmol, 0.5 equiv) was dissolved in toluene (2 mL) and **4** (45 mg, 0.12 mmol, 1 equiv) dissolved in toluene (3 mL) was added slowly. The yellow solution turned orange-brown. The solution was stirred for 4 h at ambient temperature and the solvent removed in vacuo. The orange-brown solid was washed with *n*-hexane (3 x 1.5 mL) and diethylether (1 mL) and dried *in vacuo*. **6** was obtained as an orange-brown solid (58 mg, 84%).

Crystals suitable for X-ray diffraction analysis were obtained by cooling a saturated solution of **6** in diethylether at -40 °C.

^{1}H-NMR ($CDCl_3$, 300 MHz): δ= 4.52-4.39 and 4.31-4.18 (2 x m, 2 x 2H, C2/C4-N-α-CH_2), 3.63-3.37 (m, 4H, C2/C4-N-α-CH_2)), 2.71-2.44 (m, 8H, C3-(N-α-CH_2)$_2$), 1.88-1.33 (m, 24H, β-γ-CH_2) ppm.

^{13}C{^{1}H}-NMR ($CDCl_3$, 74.5 MHz): δ= 186.9 (d, $^1J_{C-Rh}$= 52 Hz, 1C, *C*O), 184.9 (d, $^1J_{C-Rh}$= 79 Hz, 1C, *C*O), 181.4 (2C, Rh-C-(*C*≈N)$_2$), 131.2 (d, $^1J_{C-Rh}$= 32 Hz, 1C, Rh-*C*), 95.0 (1C, *C3*), 50.4 (2C, C2/4-(N-α-*C*H_2)$_2$), 50.1 (2C, C2/4-(N-α-*C*H_2)$_2$), 48.8 (4C, C3-(N-α-*C*H_2)$_2$), 26.9 (4C, C3-(N-β- *C*H_2)$_2$), 23.8, 25.2, 25.6, 26.6 (8C, β-γ-*C*H2) ppm.

IR (DCM, $\bar{\nu}_{CO}$ (cm^{-1})): 2056, 1978.

EA (%): calculated for $C_{26}H_{40}ClN_4O_2Rh$: C: 53.94, H: 6.96, N: 9.68; found: C: 53.90, H: 6.935, N: 9.51.

Synthesis of 7

$(Me_3N)W(CO)_5$ (99.5 mg, 0.26 mmol, 1 equiv) was dissolved in THF (10 mL) and **3** (100 mg, 0.26 mmol, 1 equiv) dissolved in THF (5 mL) was added slowly. The orange-yellow solution was heated to 50 °C and stirred for 16 h. The solution slowly turned brownish. The solvent was removed *in vacuo* and the brown solid washed with *n*-hexane (3 x 2 mL). The raw product was recrystallized from *n*-hexane/diethylether (10:1), and dried *in vacuo*; **7** was obtained as a yellow solid (66 mg, 36 %).

Crystals suitable for X-ray diffraction analysis were obtained by diffusion of *n*-hexane into a solution of **7** in $CHCl_3$ at -40 °C.

^{1}H-NMR ($CDCl_3$, 300 MHz): δ= 4.18-4.07 and 3.65-3.50 (2 x m, 2 x 4H, C2/C4-N-α-CH_2), 2.64-2.51 (br s, 8H, C3-(N-α-C*H*2)2), 1.81-1.43 (m, 24H, β-γ-C*H*) ppm.

^{13}C{^{1}H}-NMR ($CDCl_3$, 74.5 MHz): δ= 203.4 (1C, *trans*-CO) 201.0 (4C, CO), 184.1 (2C, W-C-(*C*≈N)$_2$), 130.1 (1C, W-*C*), 97.0 (1C, *C3*), 50.9, 50.7, 50.4, 49.0, 47.9 (8C, α-*C*H_2), 26.9 C3-(N-β-*C*H2)2 , 26.6, 25.7, 25.2, 23.8 (8C, β-γ-*C*H_2) ppm.

IR (ATR, $\bar{\nu}_{CO}$ (cm^{-1})): 2047, 1942, 1888, 1866.

EA (%): calculated for $C_{29}H_{40}N_4O_5W$: C: 49.16; H: 5.69; N: 7.91 found: C: 48.70, H: 5.924, N: 7.73.

Attempted Synthesis of S1

To a suspension of tin dichloride (150 mg, 0.79 mmol, 1.58 equiv) in THF (2 mL) bis(piperidino)acetylene (100 mg, 0.502 mmol, 1 Eq.) dissolved in THF (1 mL) was added and the yellow-orange solution was stirred for 3 d which a grey precipitate appeared. The reaction mixture was filtered, and the solvent removed *in vacuo*. The grey-yellow residue was extracted with THF, filtered and the solvent removed *in vacuo*. This was repeated until no more grey solid was present. The yellow solid thus obtained was recrystallized from THF to give the raw product as a yellow solid.

Crystals suitable for X-ray diffraction analysis were obtained by diffusion of *n*-hexane into a solution of **S1** in THF.

X-Ray structure analysis

Data were recorded with diffractometers of the firm Oxford Diffraction, using monochromated Mo *Kα* or mirror-focused Cu *Kα* radiation. Absorption corrections were applied on the basis of multi-scans. All measurements were performed at 100 K.Structures were refined anisotropically on F^2 using the program SHELXL-97, except for compounds **3** and **4b**, for which SHELXL-2017 was used.[4] Hydrogen atoms were included using a riding model or rigid methyl groups. Numerical details are summarized in Table S1.

Exceptions and special features: The slightly low completeness for **2a** (95%) was caused by the loss of the crystal (presumably by a delayed phase change at low temperature, leading to cracking). Compounds **2a** and **2b** are isotypic, but this only becomes clear if the beta angle of **2b** is changed to its complementary angle. The piperidyl units at C3 in **2a** and **2b** and also at C1 in **3** are disordered and were refined over two positions (including atom C1 for **3**). In **6** the cis-carbonyl and chloride ligands show mutual substitutional disorder and were both refined over two positions. Appropriate restraints were employed to improve refinement stability, but the dimensions of disordered groups should be interpreted with caution. Structure **4b** was refined as a non-merohedral twin using the "HKLF 5" method, with the second component rotated by 180° around the *c**-axis; the relative volume of the minor component refined to 0.164(1). Because both overlapped and non-overlapped reflections are used in the refinement, the number of reflections is ill-defined; *R*(int) is not applicable. Compound **S2** formed non-merohedral twins by 180° rotation about the *c* axis; a dataset excluding all overlapped reflections proved to give much better results than the "HKLF 5" method, although the completeness is then low (92%).

Complete data have been deposited at the Cambridge Crystallographic Data Centre under the CCDC numbers 1946165-1946174. These data can be obtained free of charge from http://www.ccdc.cam.ac.uk/data_request/cif.

Table S1a. Crystallographic data for compounds **2a**, **2b**, **3**, **4a.CHCl$_3$** and **4b**.

Compound	**2a**	**2b**	**3**	**4a·CHCl$_3$**	**4b**
Formula	$C_{24}H_{40}Cl_2N_4Sn$	$C_{24}H_{40}Cl_2GeN_4$	$C_{24}H_{40}N_4$	$C_{25}H_{41}Cl_5N_4Sn$	$C_{24}H_{40}Cl_2GeN_4$
M_w [g mol^{-1}]	574.19	528.09	384.60	693.56	528.09
λ [Å]	1.54184	1.54184	1.54184	1.54184	1.54184
T [K]	100(2)	100(2)	100(2)	100(2)	100(2)
Crystal size [mm^3]	0.10 x 0.08 x 0.04	0.20 x 0.20 x 0.15	0.39 x 0.33 x 0.26	0.2 x 0.2 x 0.1	0.16 x 0.12 x 0.03
Crystal system	monoclinic	monoclinic	monoclinic	triclinic	triclinic
Space group	$P2_1/c$	$P2_1/c$	$P2_1/n$	P(-1)	P(-1)
a [Å]	9.4071(4)	9.2768(2)	13.85628(16)	11.1344(6)	8.8038(4)
b [Å]	13.9126(6)	13.9144(2)	11.63685(14)	11.9045(8)	10.1672(4)
c [Å]	20.0014(9)	19.8673(4)	14.32768(18)	13.0371(6)	14.9878(6)
α [°]	90	90	90	93.803(4)	79.153(4)
β [°]	90.663(4)	90.453(2)	104.9367(14)	90.663(4)	78.743(4)
γ [°]	90	90	90	113.546(6)	74.761(4)
V [Å^3]	2617.5	2564.41	2232.18	1494.21	1256.13
Z	4	4	4	2	2
ρ_{calc} [Mg m^{-3}]	1.457	1.368	1.144	1.542	1.396
μ [mm^{-1}]	9.8	3.7	0.52	11.1	3.7
F (000)	1184	1112	848	708	556
Reflections collected	20106	89868	45113	50261	6436
Indep. reflections (R_{int})	5146 (0.054)	5339 (0.032)	4658 (0.045)	6174 (0.044)	6436
Restraints / parameters	72 / 301	66 / 301	58 / 282	0 / 316	0 / 281
Completeness to ϑ [%] (ϑ)	94.9 (75.0°)	99.9 (75.0°)	99.4 (76.3°)	99.8 (75.0°)	99.9 (75.0°)
Goodness of Fit on F^2	1.02	1.07	1.03	1.05	1.05
R_1 ($I>2\sigma(I)$)	0.0332	0.0221	0.0463	0.0240	0.0425
wR_2 (all refl.)	0.0816	0.0557	0.1277	0.0603	0.1218
Theta range [°]	3.9-76.0	3.9-76.0	3.9-76.3	3.6-76.0	4.6-76.2
max. Δp [e Å^{-3}] max/min	0.56/-0.94	0.28/-0.39	0.44/-0.19	1.07/-0.76	0.86/-1.03
CCDC	1946165	1946166	1946167	1946168	1946169

Table S1b. Crystallographic data for compounds **5.CH_2Cl_2**, **6**, **7**, **S1** and **S2**.

Compound	**5·CH_2Cl_2**	**6**	**7**	**S1**	**S2**
Formula	$C_{25}H_{42}AuCl_3N_4$	$C_{26}H_{40}ClN_4O_2Rh$	$C_{29}H_{40}N_4O_5W$	$C_{24}H_{40}Cl_6N_4Sn_2$	$C_{12}H_{20}Cl_6Ge_2N_2$
M_w [g mol^{-1}]	701.94	578.98	708.50	834.68	550.18
λ [Å]	0.71073	0.71073	0.71073	0.71073	1.54184
T [K]	100(2)	100(2)	100(2)	100(2)	100(2)
Crystal size [mm^3]	0.20 x 0.18 x 0.14	0.28 x 0.22 x 0.12	0.18 x 0.16 x 0.10	0.18 x 0.16 x 0.12	0.22 x 0.06 x 0.04
Crystal system	monoclinic	triclinic	monoclinic	monoclinic	monoclinic
Space group	$P2_1/n$	P(-1)	$P2_1/n$	$P2_1/n$	P2$_1$/c
a [Å]	14.1149(4)	10.3912(4)	12.1126(4)	11.8931(3)	9.6767(4)
b [Å]	11.5638(5)	12.0730(5)	14.0607(4)	15.1698(3)	8.6266(3)
c [Å]	17.2843(6)	12.9292(5)	17.0864(4)	17.8116(4)	12.3673(6)
α [°]	90	106.605(4)	90	90	90
β [°]	98.160(3)	102.506(4)	96.144(3)	94.106(2)	104.357(5)
γ [°]	90	112.450(4)	90	90	90
V [Å^3]	2792.60	1335.91	2893.29	3205.26	1000.15
Z	4	2	4	4	2
ρ_{calc} [Mg m^{-3}]	1.670	1.439	1.627	1.730	1.827
μ [mm^{-1}]	5.6	0.77	4.0	2.1	11.1
F (000)	1400	604	1424	1656	544
Reflections collected	72680	71675	75448	169671	18151
Indep. reflections (R_{int})	8440 (0.078)	8004 (0.038)	8371 (0.082)	9898 (0.059)	1917 (0.066)
Restraints / parameters	0 / 298	29 / 320	0 / 352	0 / 325	0 / 100
Completeness to ϑ [%] (ϑ)	99.2 (30.0°)	98.9 (30.0°)	99.1 (30.0°)	99.0 (30.5°)	92.2 (75.0°)
Goodness of Fit on F^2	1.05	1.09	1.05	1.10	1.10
R_1 ($I>2\sigma(I)$)	0.0383	0.0261	0.0315	0.0339	0. 0309
wR_2 (all refl.)	0.0877	0.0554	0.0627	0.0767	0.0777
Theta range [°]	2.3-31.1	2.2-31.1	2.2-30.0	2.3-61.2	4.7-76.1
max. Δp [e Å^{-3}] max/min	1.73/-1.34	0.55/-0.42	1.75/-1.16	1.05/-0.85	0.48 /-0.75
CCDC	1946170	1946171	1946172	1946173	1946174

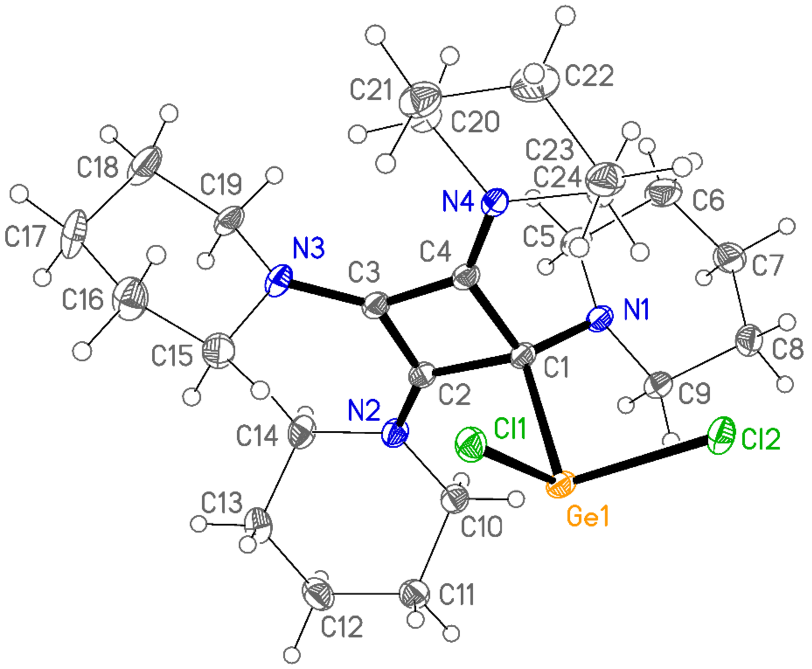

Fig. S1: Structure of **2b** with thermal displacement parameters drawn at the 50 % probability level. A disordered position of the piperidyl ring at C3 has been omitted for clarity.

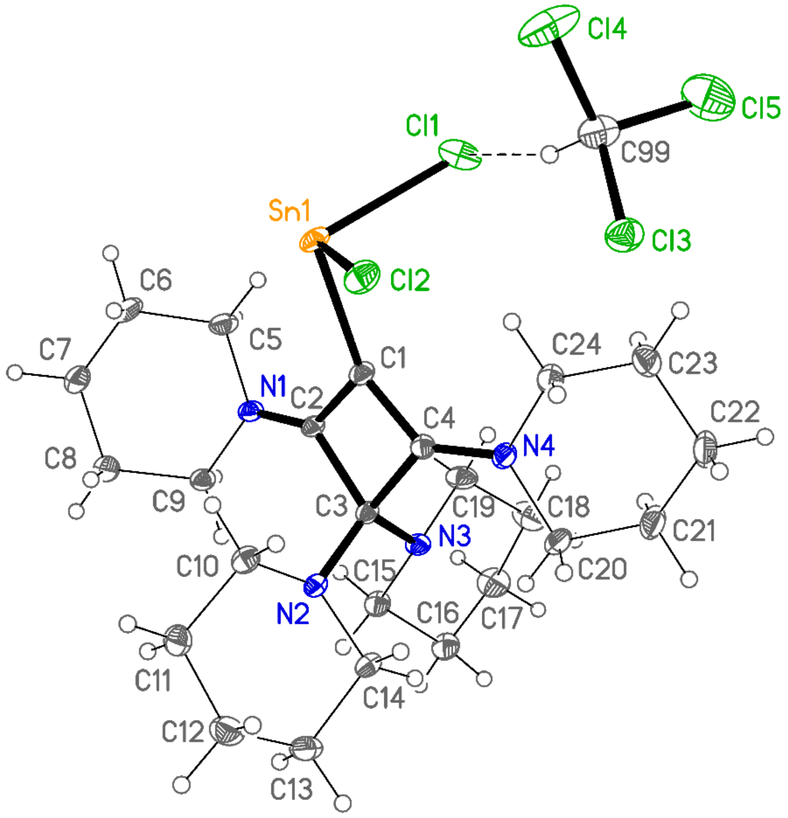

Fig. S2: Structure of **4a·CHCl$_3$**, including the solvent molecule, with thermal displacement parameters drawn at the 50 % probability level. The dashed line indicates a C···Cl hydrogen bond, with H…Cl 2.60 Å.

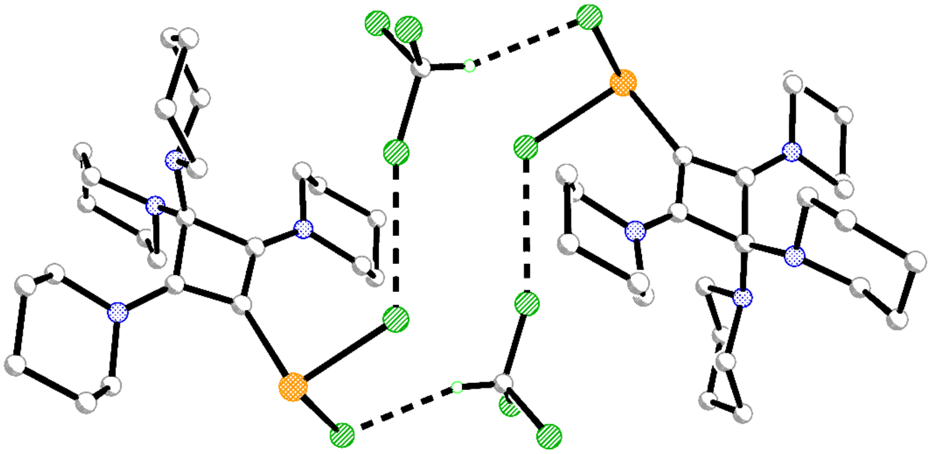

Fig. S3: Extended structure of **4a·CHCl$_3$**, showing the formation of inversion-symmetric dimers via the C–H···Cl hydrogen bond and a Cl2···Cl3 contact of 3.33 Å.

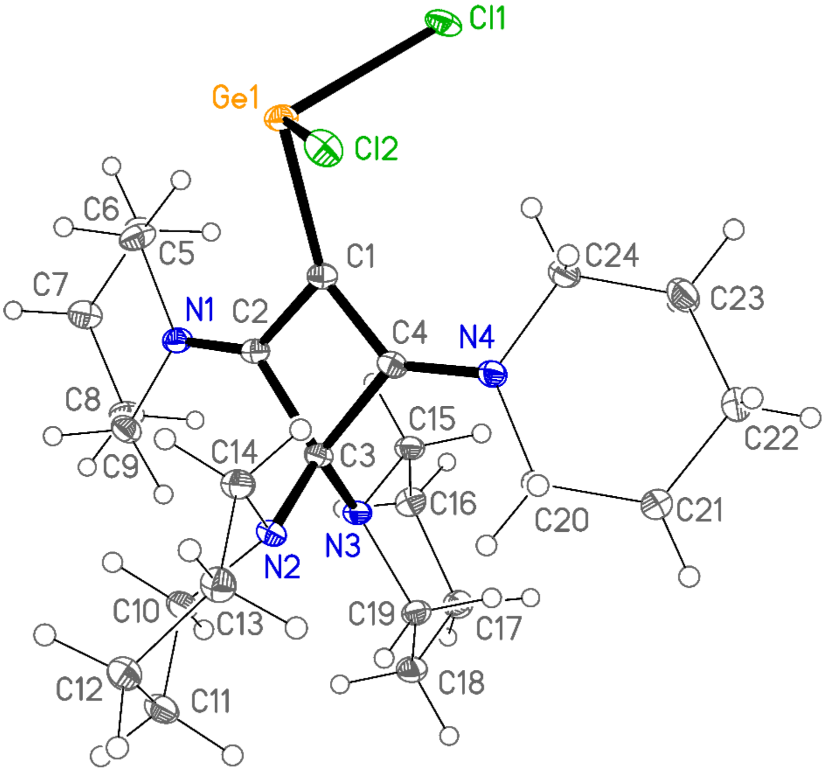

Fig. S4: Structure of **4b** with thermal displacement parameters drawn at the 30 % probability level.

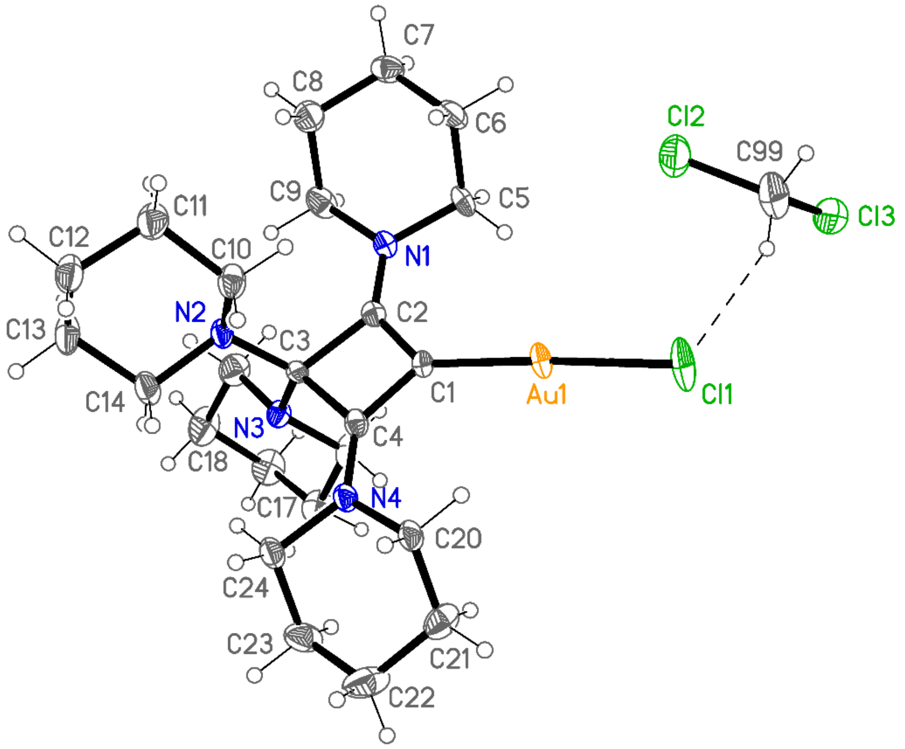

Fig. S5: Structure of **5·CH_2Cl_2**, including the solvent molecule, with thermal displacement parameters drawn at the 50 % probability level.

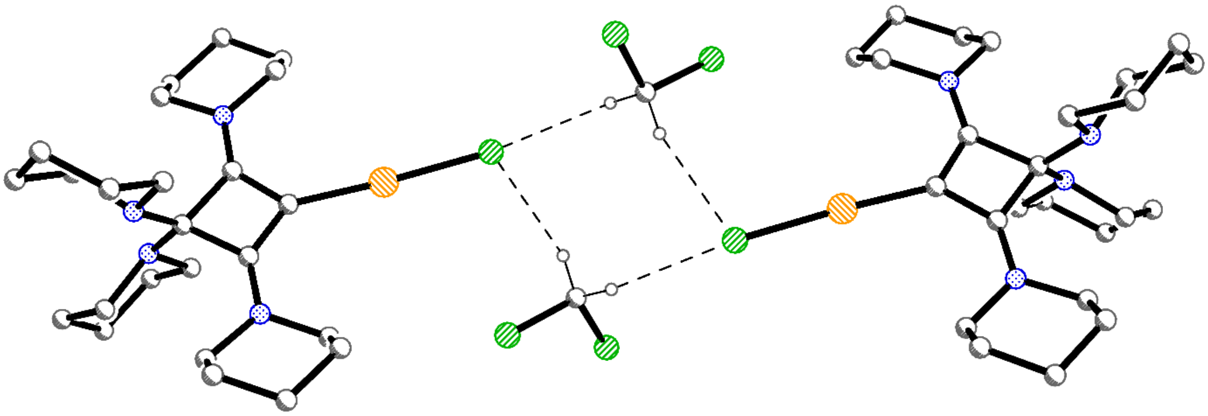

Fig. S6: Extended structure of **5·CH_2Cl_2**, showing the formation of inversion-symmetric dimers via two independent (four in total) C–H...Cl hydrogen bonds with H···Cl 2.64, 2.70 Å.

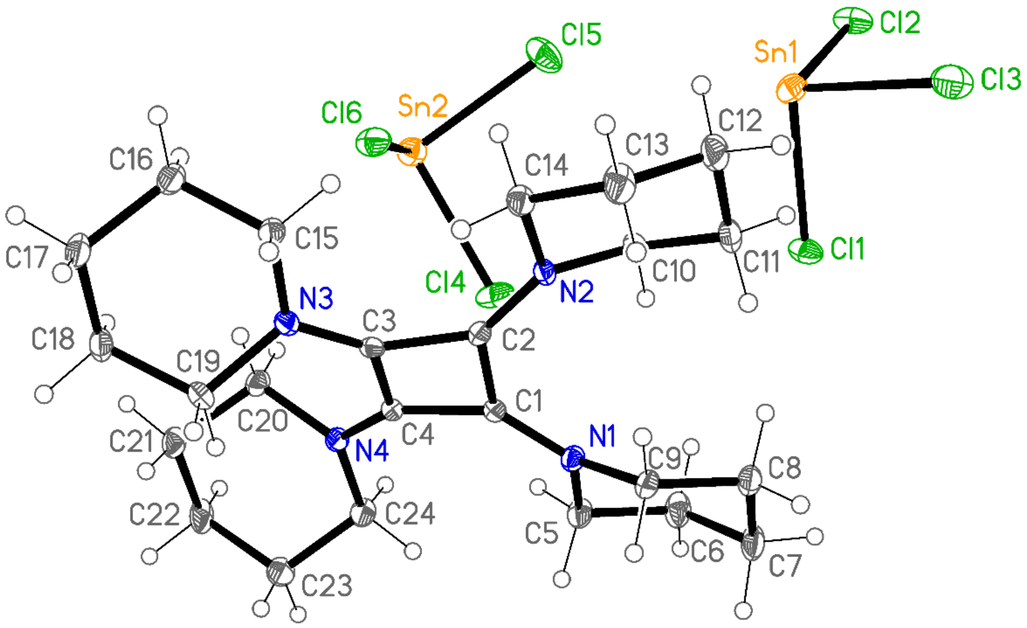

Fig. S7: The cyclobutadiene dication salt **S1** with thermal displacement parameters drawn at the 50 % probability level. Selected bond lengths [Å] and angles [°]: C1–C2 1.437(3), C1–C4 1.460(3), C2–C3 1.465(3), C3–C4 1.448(3), C1–N1 1.324(3), C2–N2 1.319(3), C3–N3 1.313(3), C4–N4 1.320(3), C1–C2–C3 89.85(18), C2–C3–C4 88.97(18), C3–C4–C1 89.64(18), C4–C1–C2 89.57(18). Angle between C4–C1–C2 and C4–C3–C2 plane: 15.0(3)°.

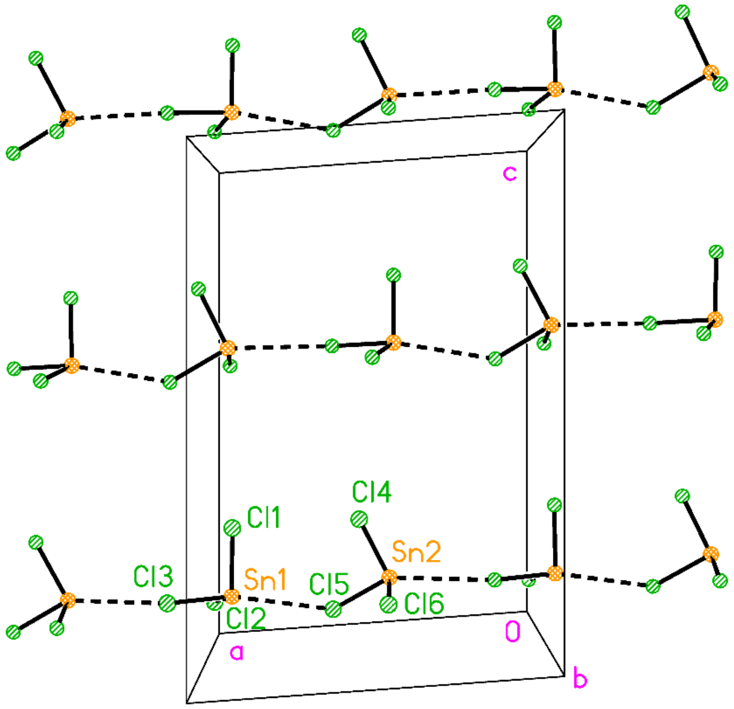

Fig. S8: Chains of trichlorostannate(II) anions parallel to the *a* axis in the structure of the cyclobutadiene dication salt **S1**.The contact distances are Sn1···Cl5 3.8472(8) and Sn2···Cl3' (operator -1+*x*, *y*, *z*) 4.0411(10) Å.

The solid-state structure of **S1** reveals that the four-membered-ring is not planar, as might be expected in view of its Hückel aromaticity, but instead shows a puckered geometry with an angle between the C2—C1—C4 and C2—C3—C4 planes of 15.0(3)°. This structure of the cyclobutadiene dication was discussed for the tetramethylcyclobutadiene dication prepared in SbF_5—SO_2 at -78°C by Olah *et al.* in 1969, who concluded a C_{4v} symmetry based on the NMR spectra.[5] This geometry was questioned by Schleyer *et al.*, who performed computational investigations on $C_4H_4^{2+}$ and its tetramethylated derivative.[6] Based on these calculations, the puckered geometry (C_{2v}) should indeed be favoured over the planar geometry, mostly because of the increased stabilising orbital mixing. Further theoretical investigations utilising various methods have emphasized the greater stability of the puckered form for several differently substituted derivatives, except for $C_4F_4^{2+}$ and $C_4(CN)_4^{2+}$.[7] The puckering has also been linked to a pseudo-Jahn-Teller effect in more recent work.[8] This is reflected by the carbon-nitrogen bond lengths which, with an average length of 1.319 Å, lie between the expected values for a single and a double bond.

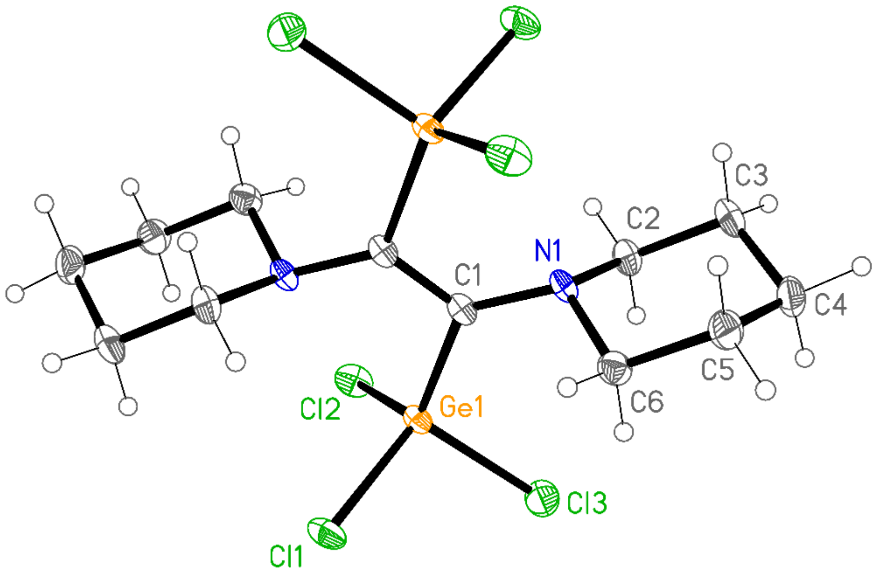

Fig. S9: Structure of **S2** with thermal displacement parameters drawn at the 50 % probability level. Only the asymmetric unit is numbered; the molecule is completed via inversion (1–*x*, 1–*y*, 1–*z*). Selected bond lengths [Å] and angles [°]: Ge1–C1 1.965(2), C1–C1' 1.330(5), Ge1–Cl1 2.1338(6), Ge1–Cl2 2.1303(6), Ge1–Cl3 2.1557(7), C1–N1 1.414(3), C1–Ge1–Cl2 117.42(7), C1–Ge1–Cl1 114.56(7), C1–Ge1–Cl3 109.10(7), Cl2–Ge1–Cl3 102.23(3), Cl2–Ge1–Cl1 108.62(3), Cl1–Ge1–Cl3 103.26(3), C1'–C1–N 120.7(3) [Symmetry related atoms are indicated by a prime].

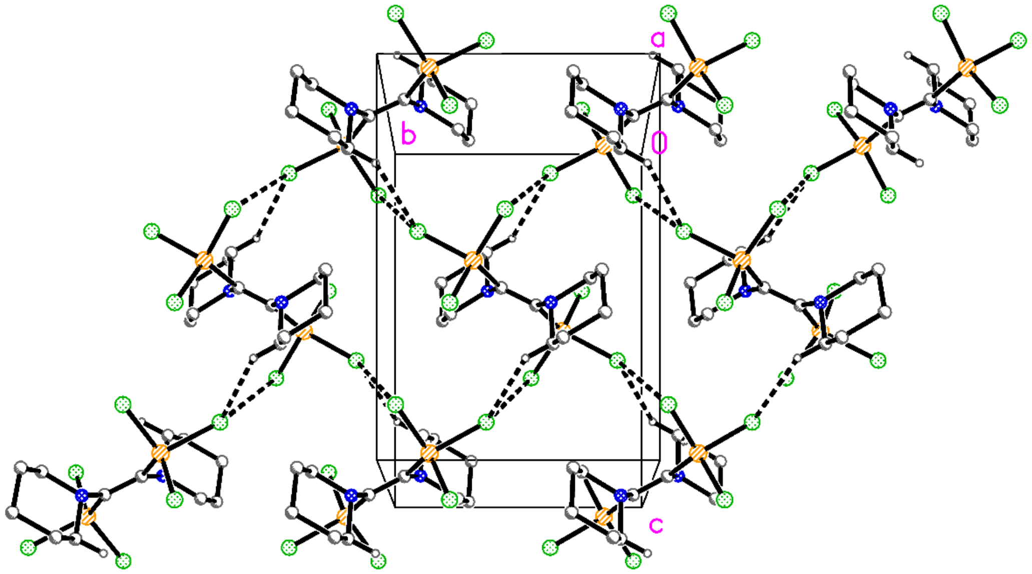

Fig. S10: Packing diagram of **S2**. Molecules are connected to form layers parallel to the *bc* plane by the contacts H2A···Cl3 2.88 Å and Cl2···Cl3 3.539 Å (operator 1–*x*, -0.5–*y*, 0.5–*z*).

NMR-spectra

NMR-Spectra of 2a

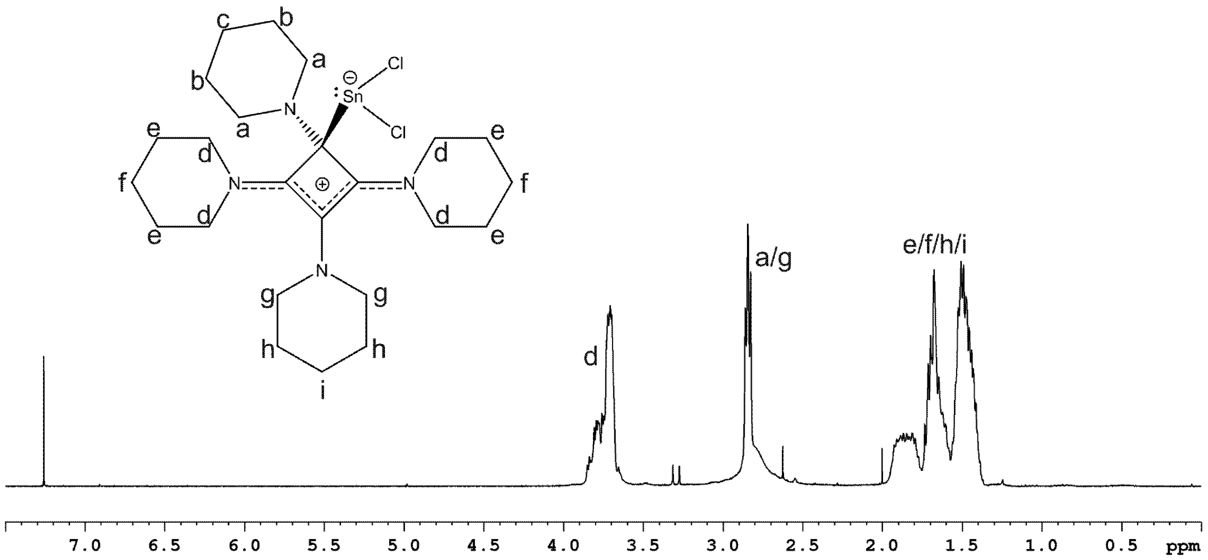

Fig. S10: ^{1}H-NMR ($CDCl_3$, 300 MHz, 300K) of **2a.**

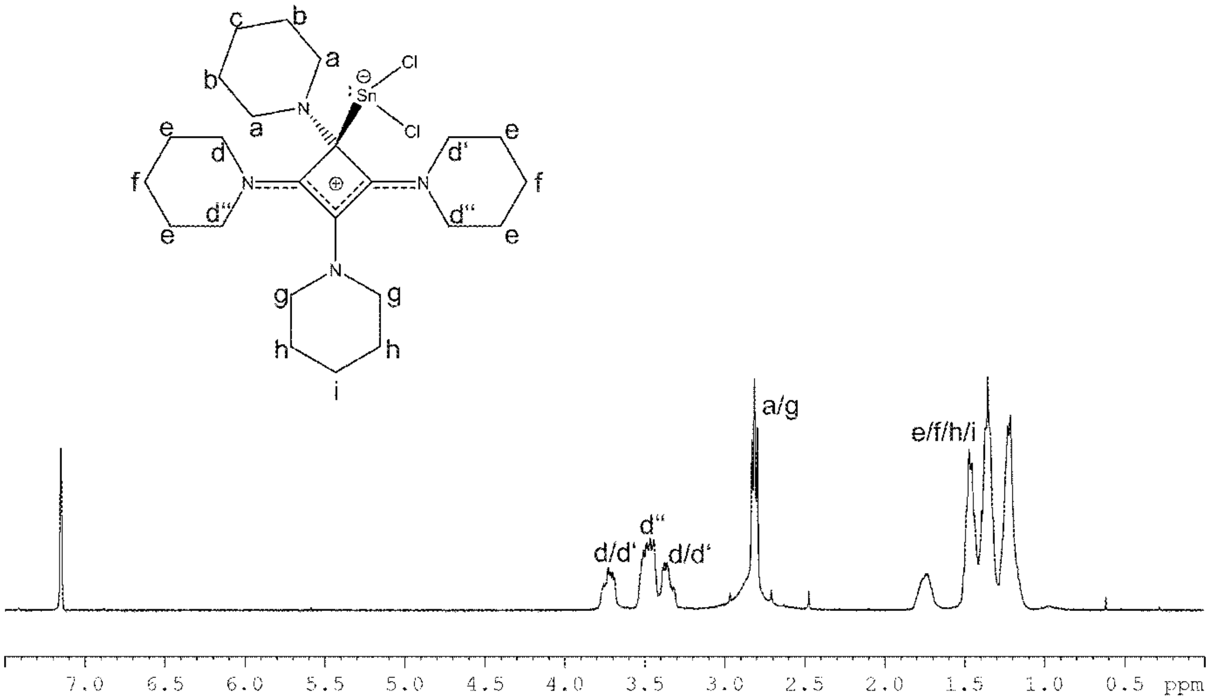

Fig. S11: ^{1}H-NMR (C_6D_6, 300 MHz, 300K) of **2a.**

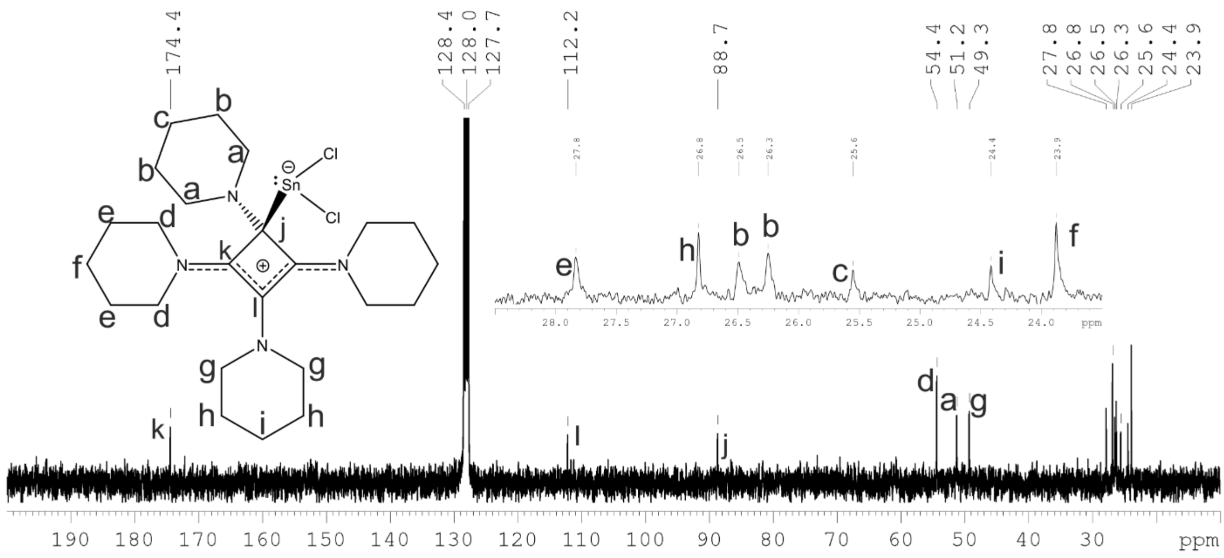

Fig. S12: $^{13}C\{^{1}H\}$-NMR (C_6D_6, 75.1 MHz, 300K) of **2a.**

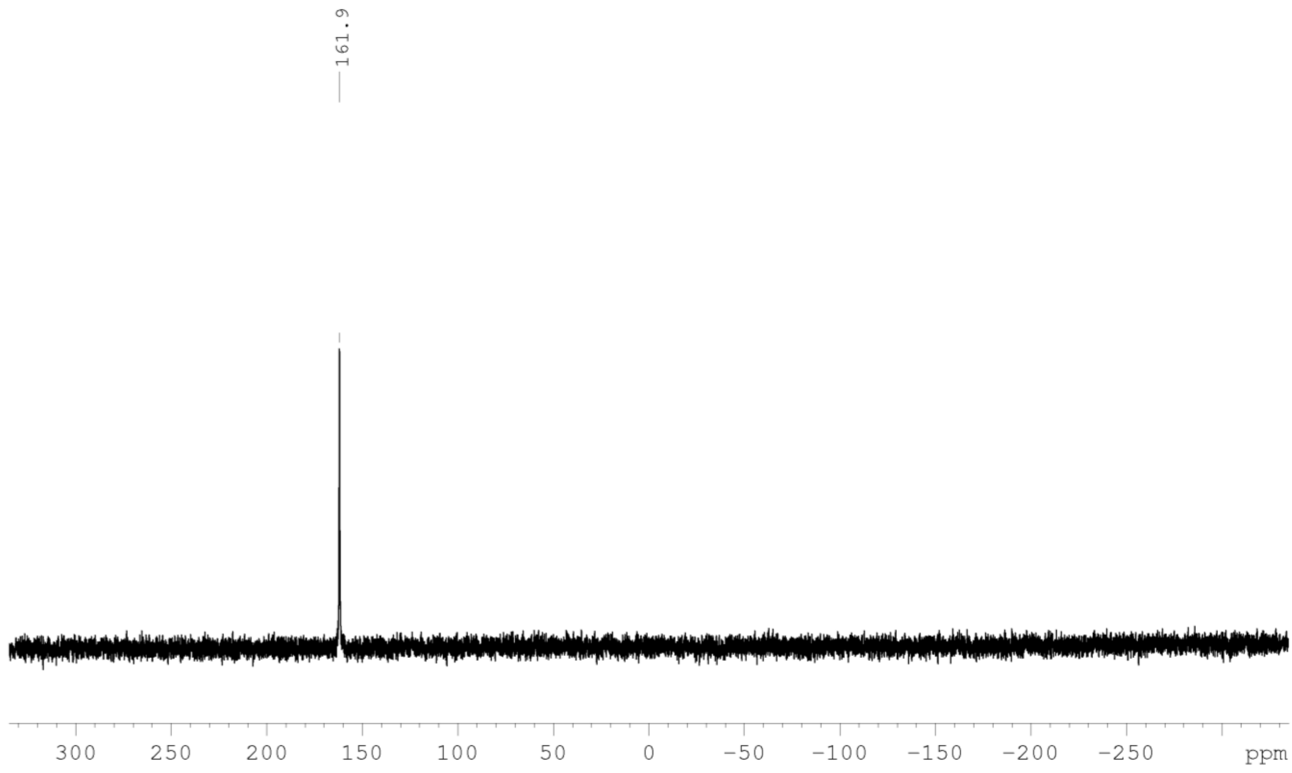

Fig. S13: $^{119}Sn\{^{1}H\}$-NMR ($CDCl_3$, 149.3 MHz, 300K) of **2a**.

NMR-Spectra of 2b

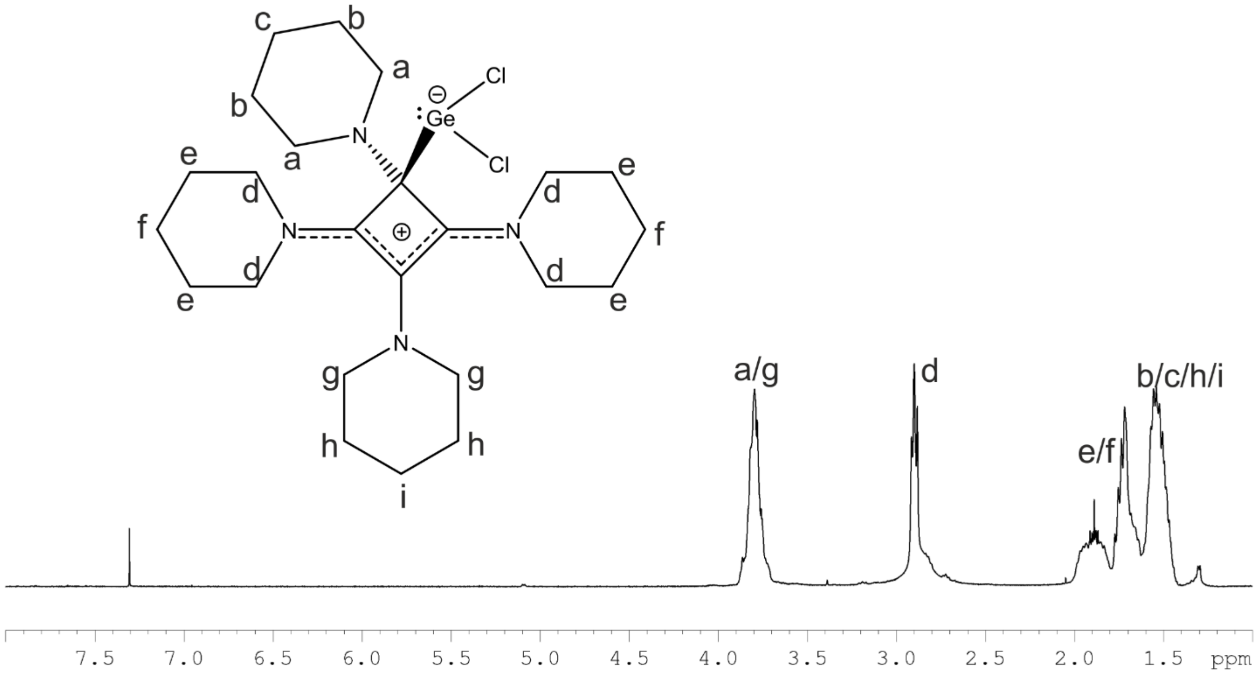

Fig. S14: ^{1}H-NMR ($CDCl_3$, 300 MHz, 300K) of **2b.**

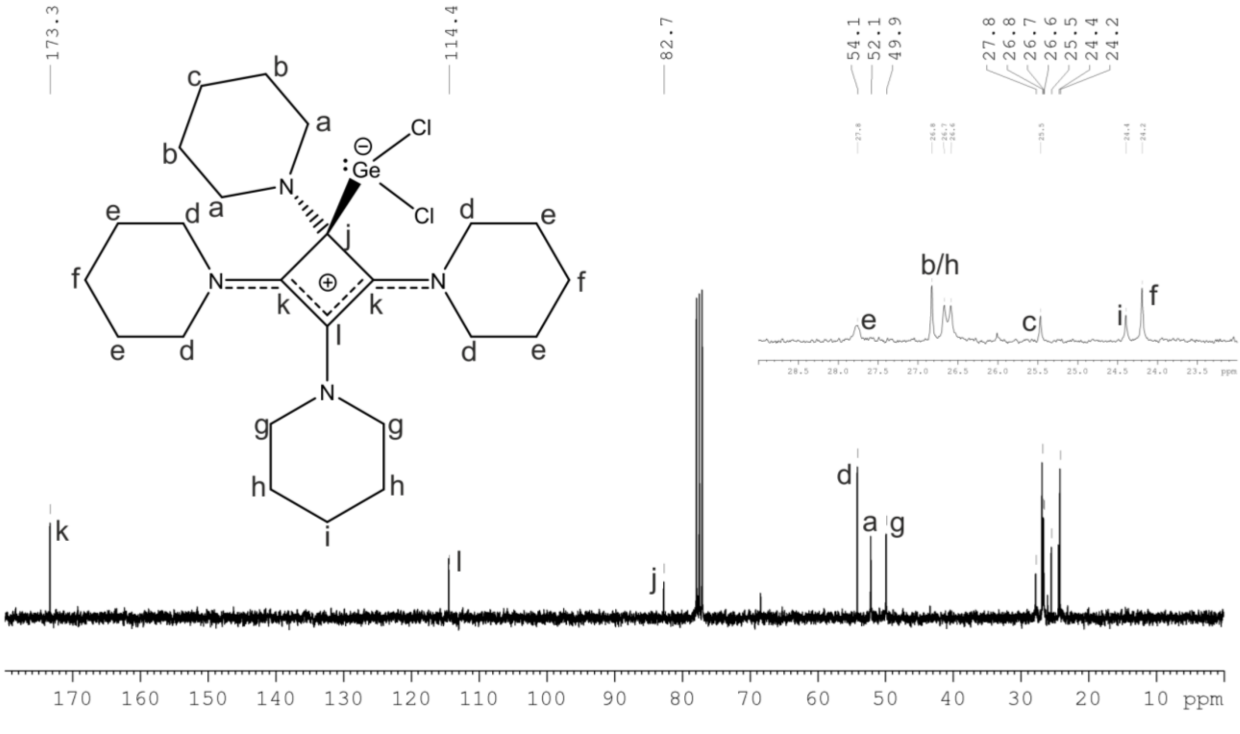

Fig. S15: ^{13}C{^{1}H}-NMR ($CDCl_3$, 75.1 MHz, 300K) of **2b**

NMR-Spectra of 3

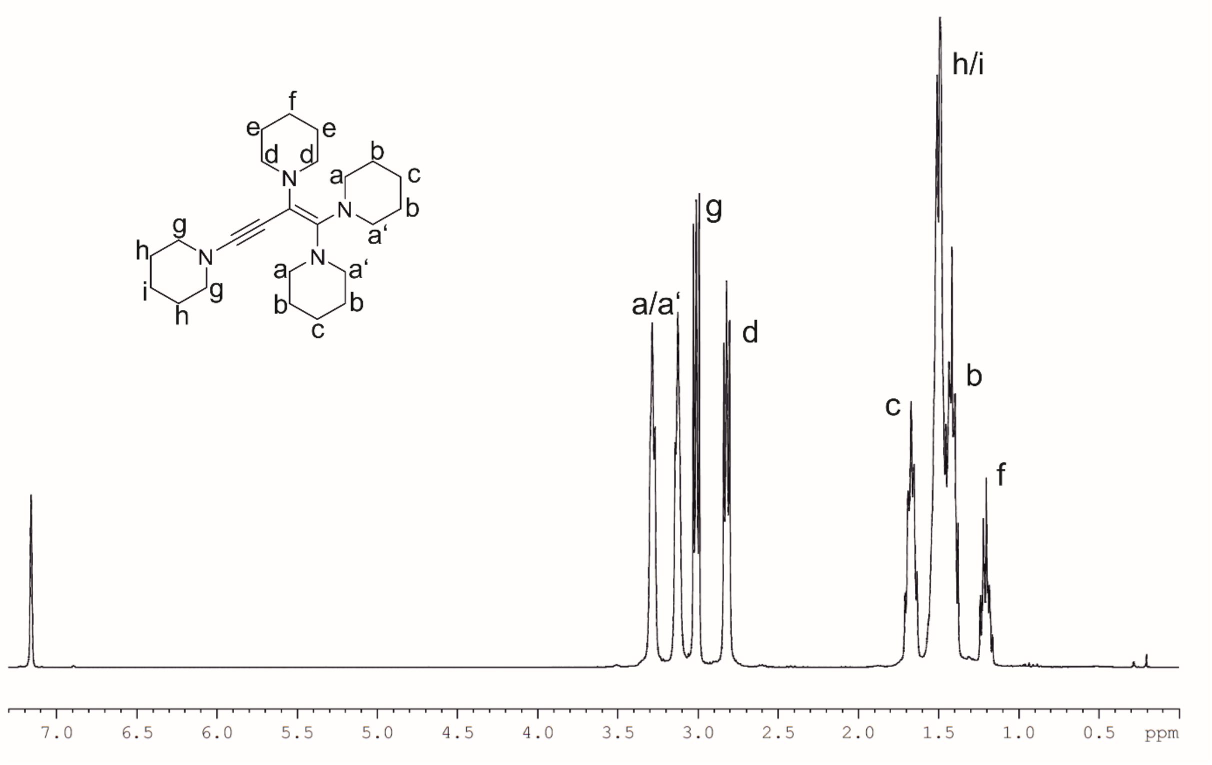

Fig. S16: ^{1}H-NMR (C_6D_6, 300 MHz, 300K) of **3**.

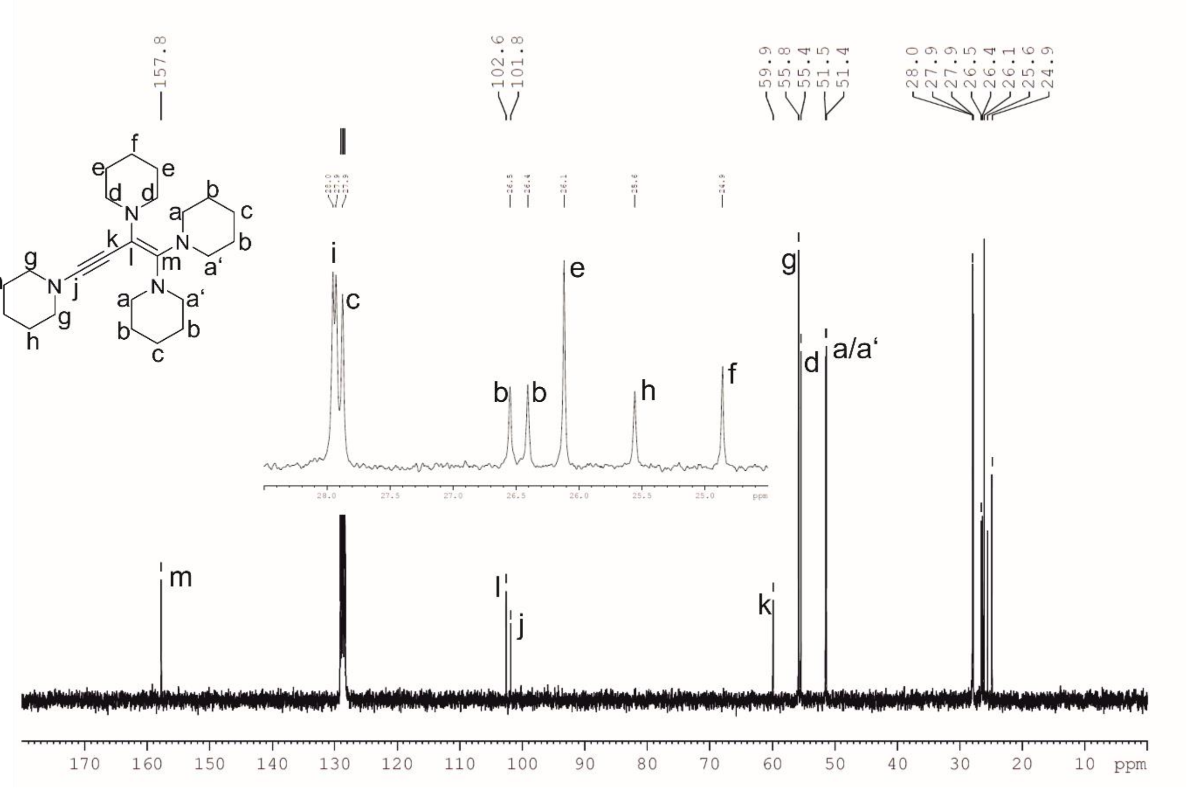

Fig. S17: ^{13}C{^{1}H}-NMR (C_6D_6, 75.1 MHz, 300K) of **3**.

NMR-Spectra of 4a

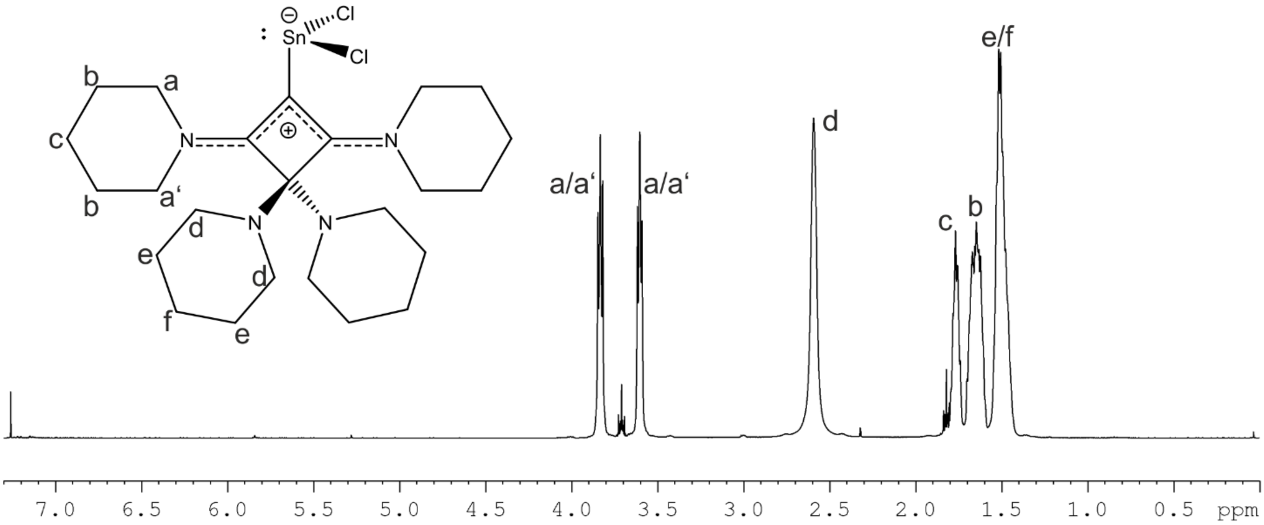

Fig. S18: ^{1}H-NMR ($CDCl_3$, 400 MHz, 300K) of **4a**.

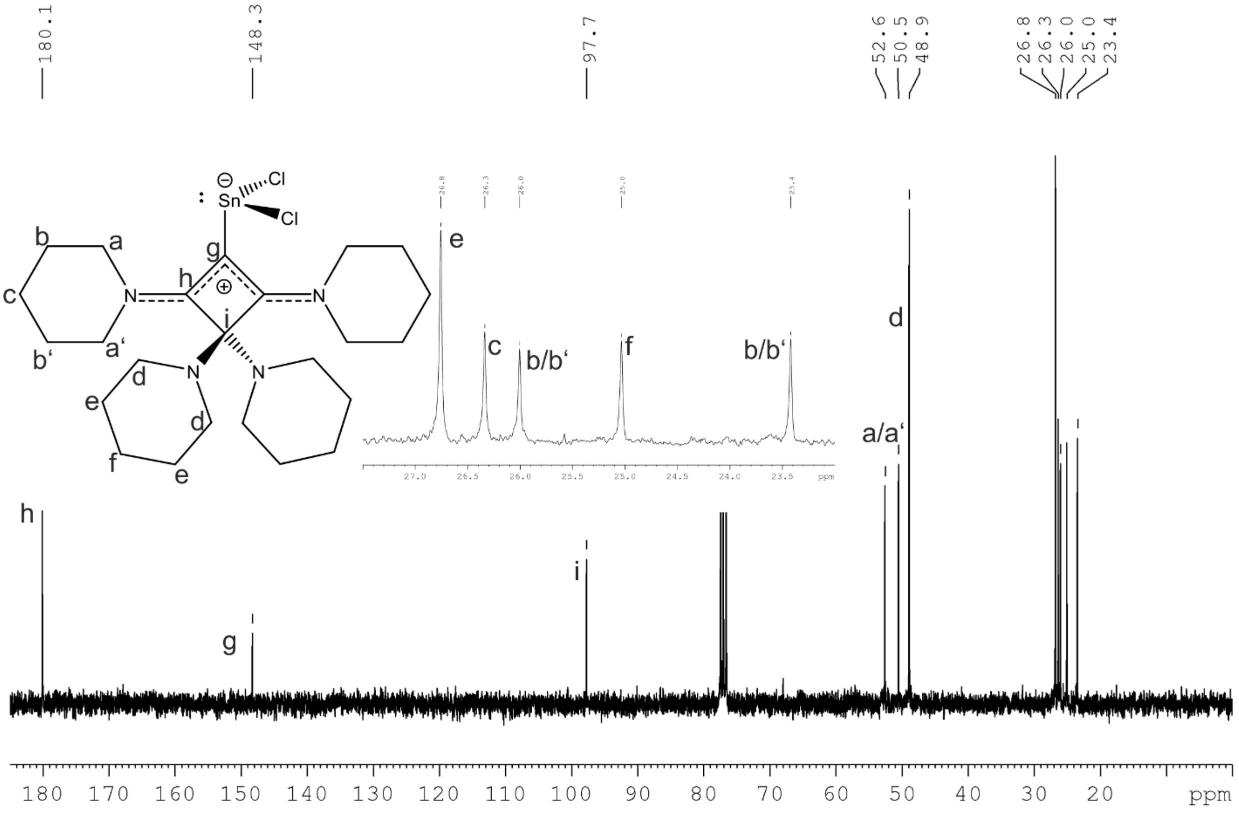

Fig. S19: ^{13}C{^{1}H}-NMR ($CDCl_3$, 100.7 MHz, 300K) of **4a**.

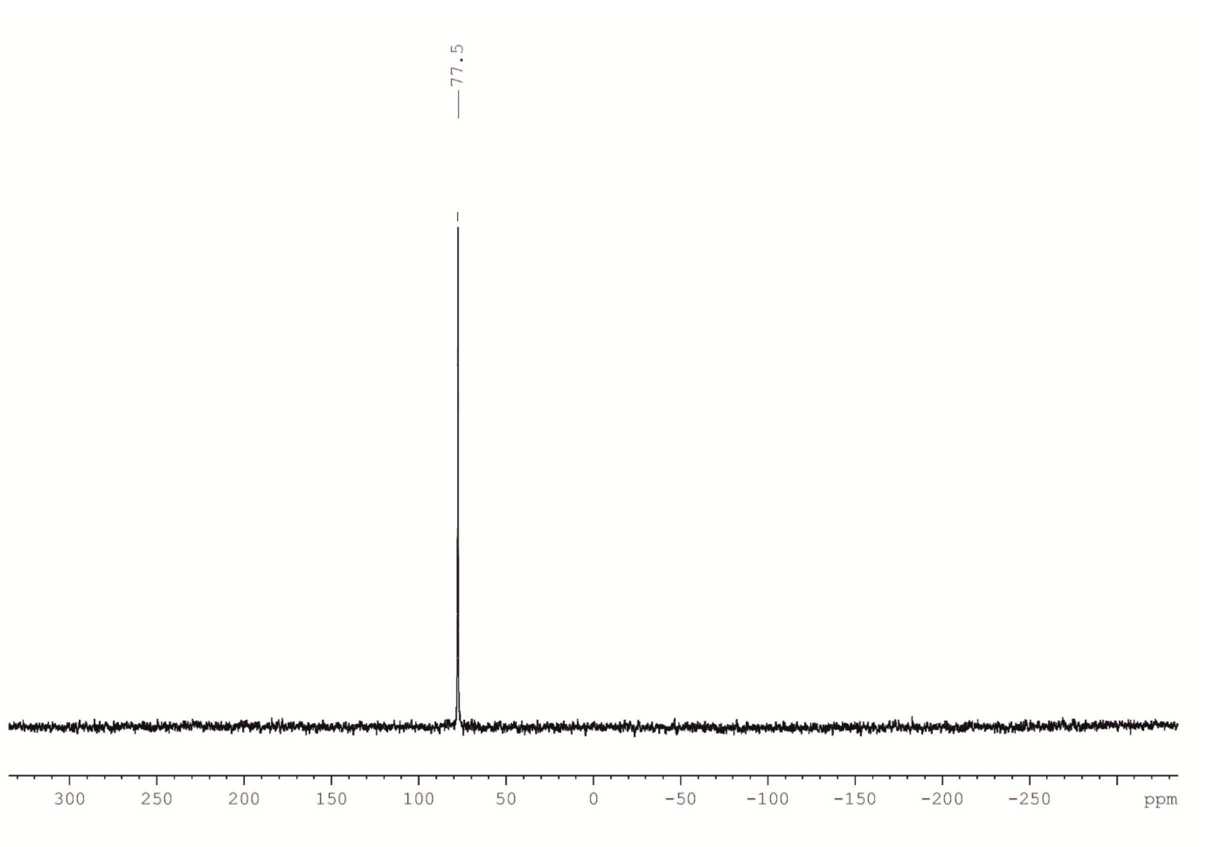

Fig. S20: $^{119}Sn\{^{1}H\}$-NMR ($CDCl_3$, 149.3 MHz, 300K) of **4a**.

NMR-Spectra of 4b

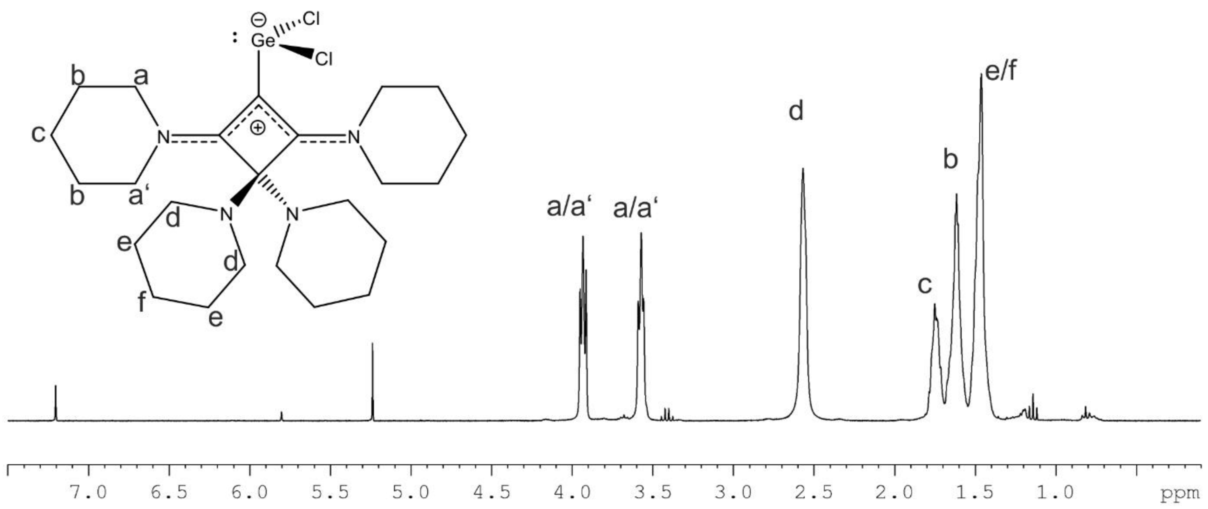

Fig. S21: ^{1}H-NMR ($CDCl_3$, 300 MHz, 300K) of **4b**.

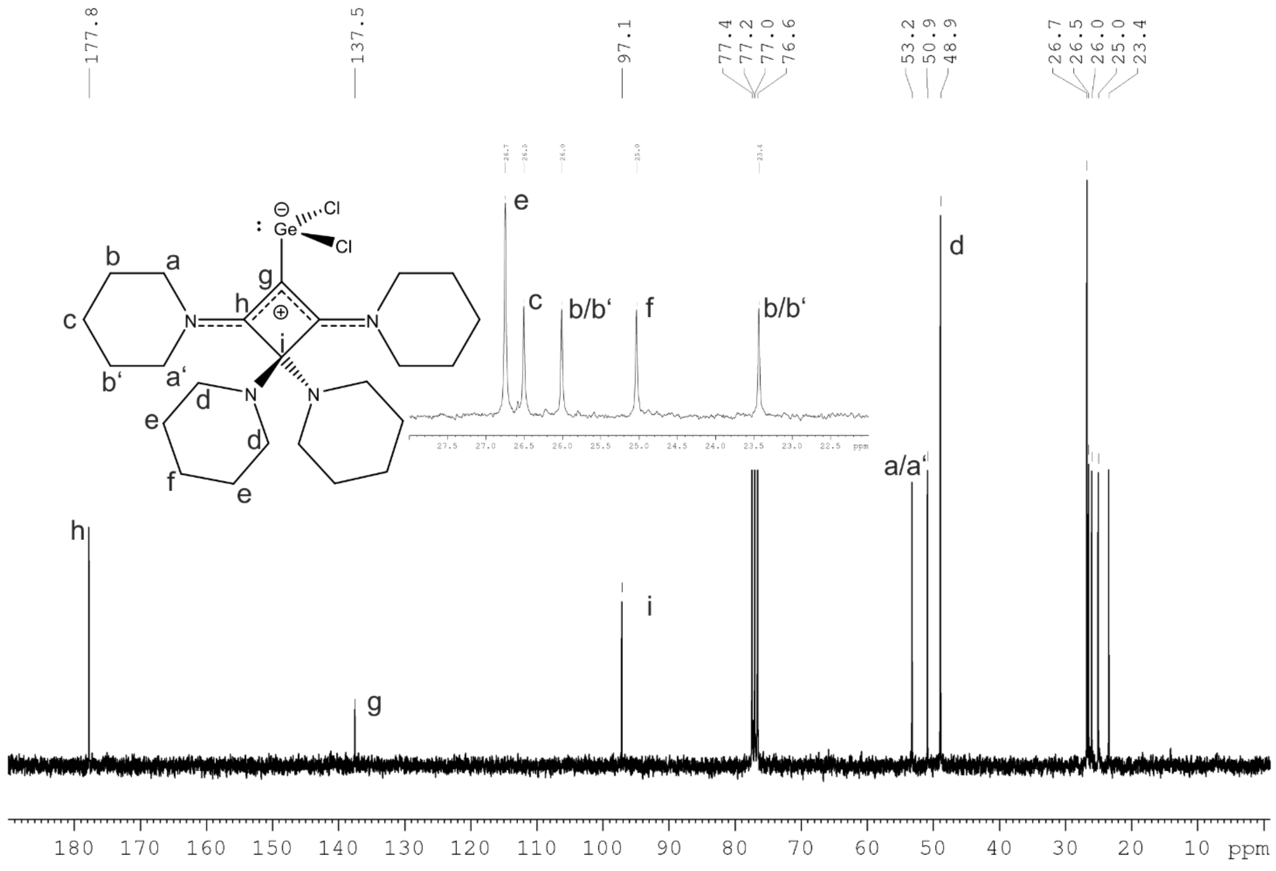

Fig. S22: ^{13}C{^{1}H}-NMR ($CDCl_3$, 75.5 MHz, 300K) of **4b**.

NM1R-Spectra of 5

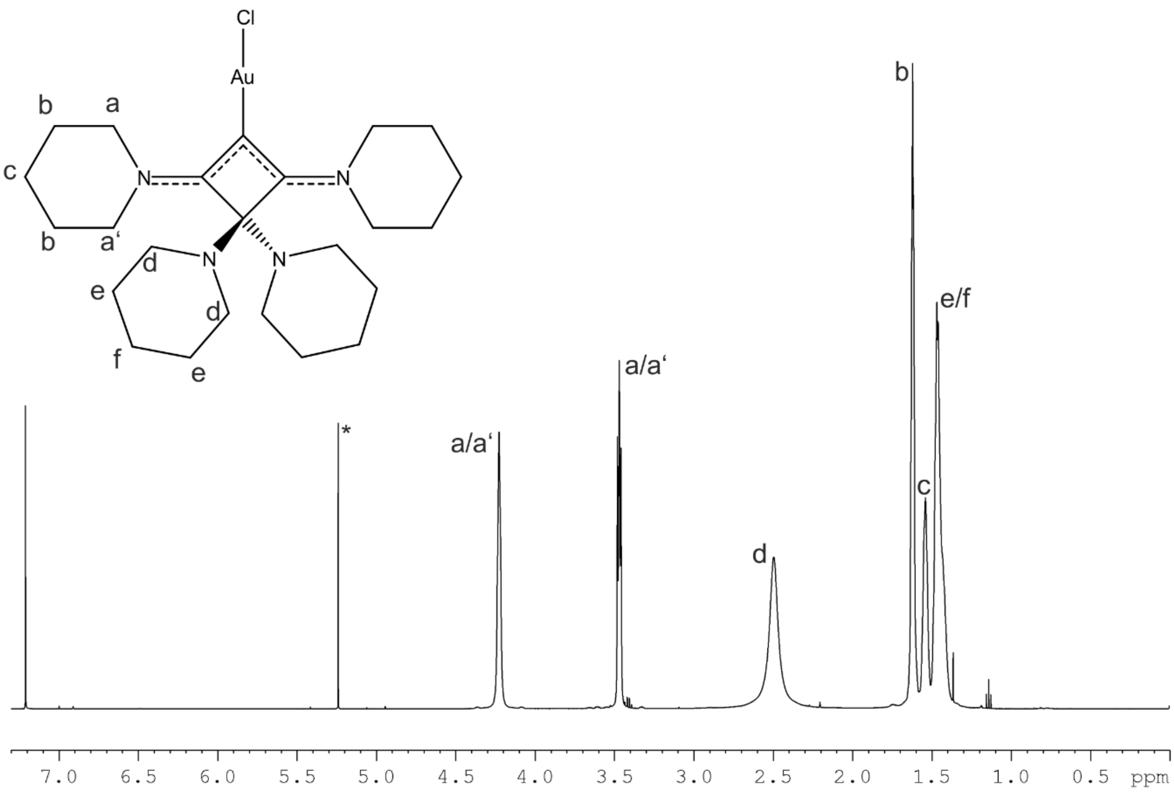

Fig. S23: ^{1}H-NMR ($CDCl_3$, 500 MHz, 300K) of **5**; * = residual DCM.

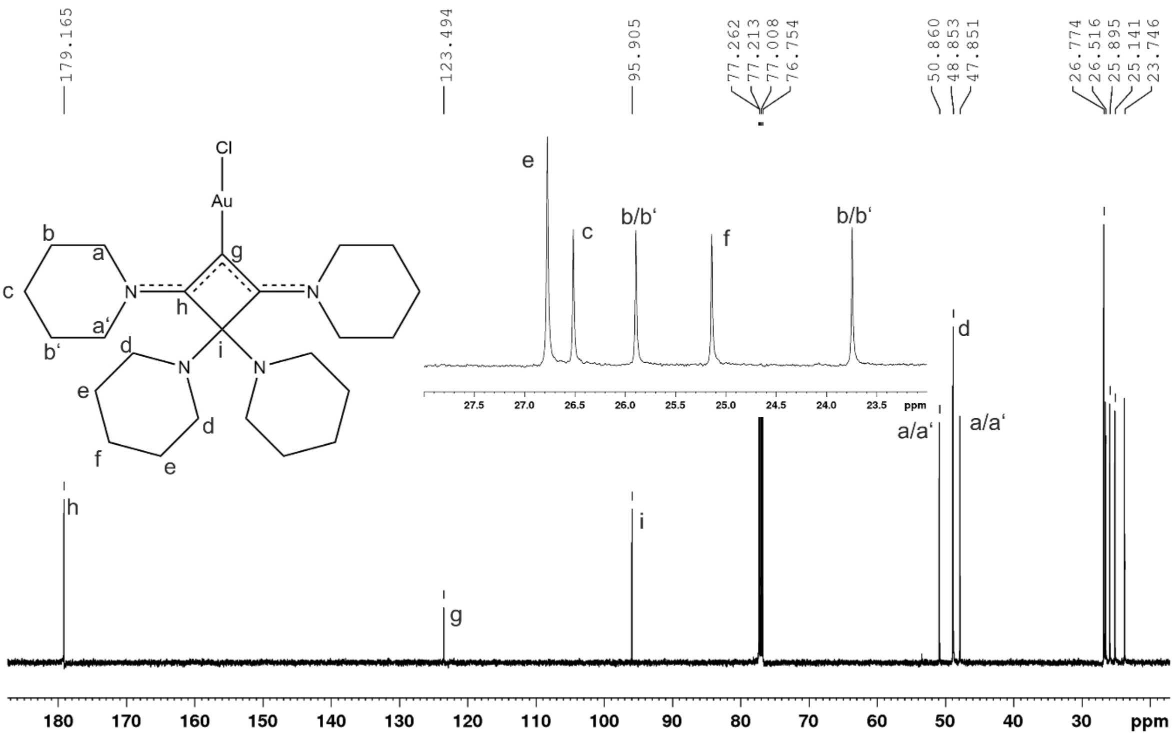

Fig. S24: $^{13}C\{^{1}H\}$-NMR ($CDCl_3$, 125.8 MHz, 300K) of **5.**

NMR-Spectra of 6

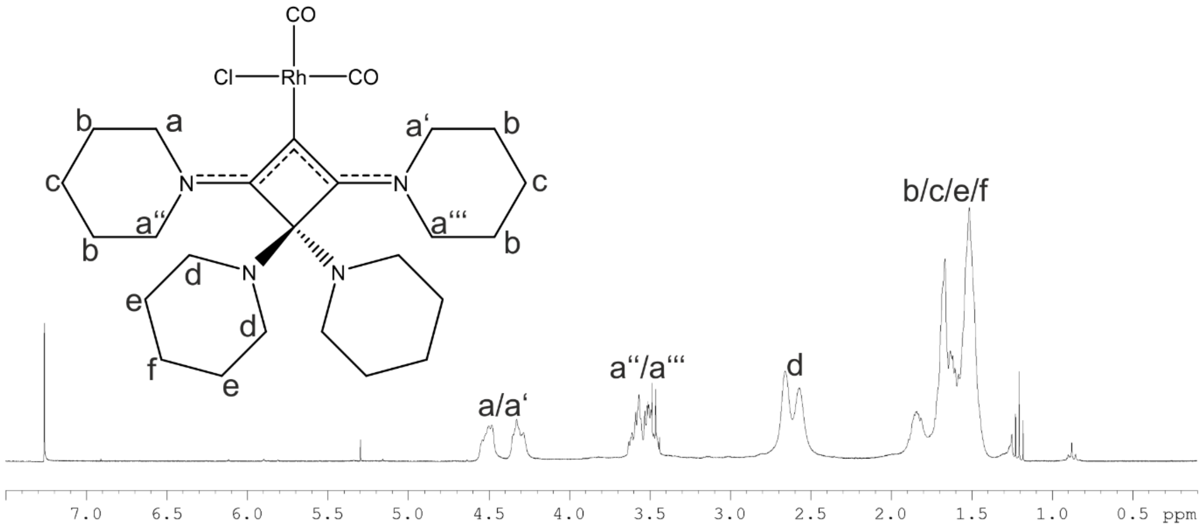

Fig. S25: ^{1}H-NMR ($CDCl_3$, 500 MHz, 300K) of **6**;

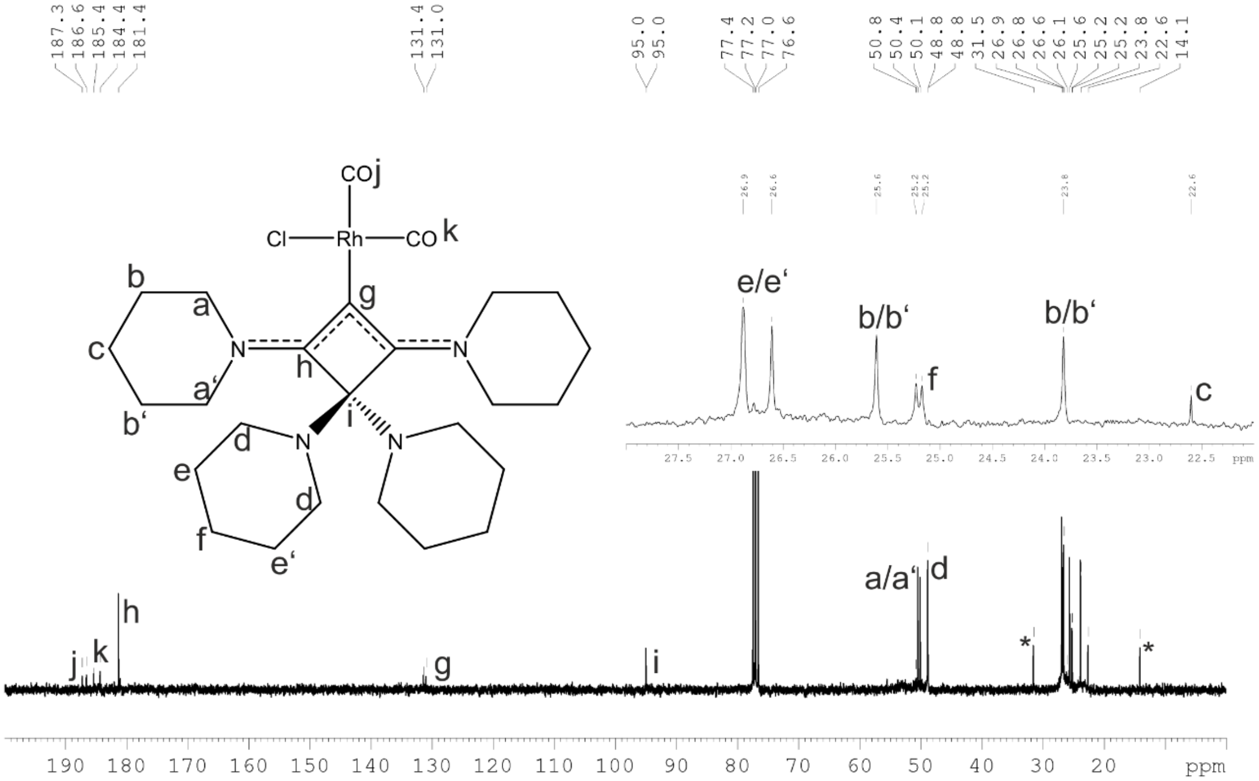

Fig. S26: $^{13}C\{^{1}H\}$-NMR ($CDCl_3$, 125.8 MHz, 300K) of **6.** * = residual Et_2O.

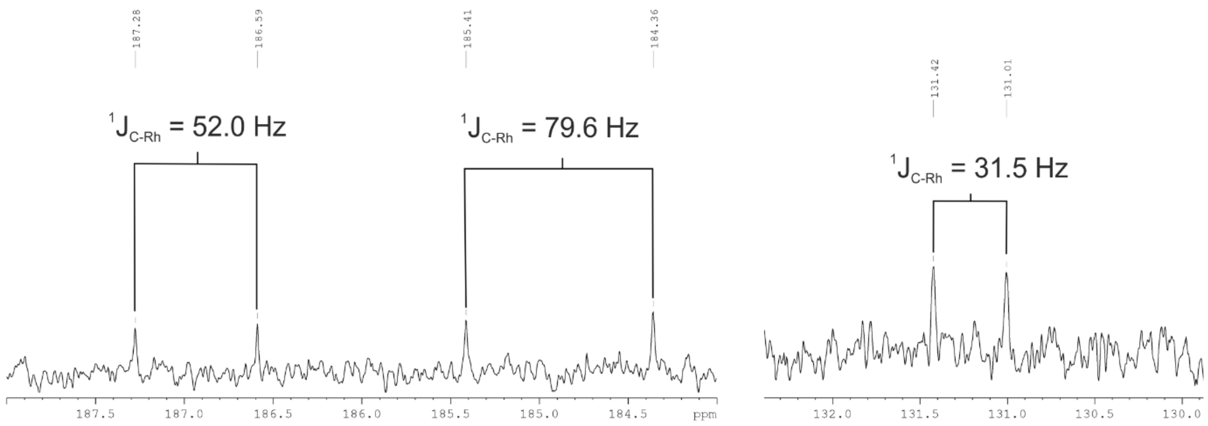

Fig. S27: ^{13}C-{^{1}H}-NMR ($CDCl_3$, 75.5 MHz, 300K) of **6**, Enlargement of the Carbonyl (left) and the central CBA-carbon (**g**) region (right).

NMR-Spectra of 7

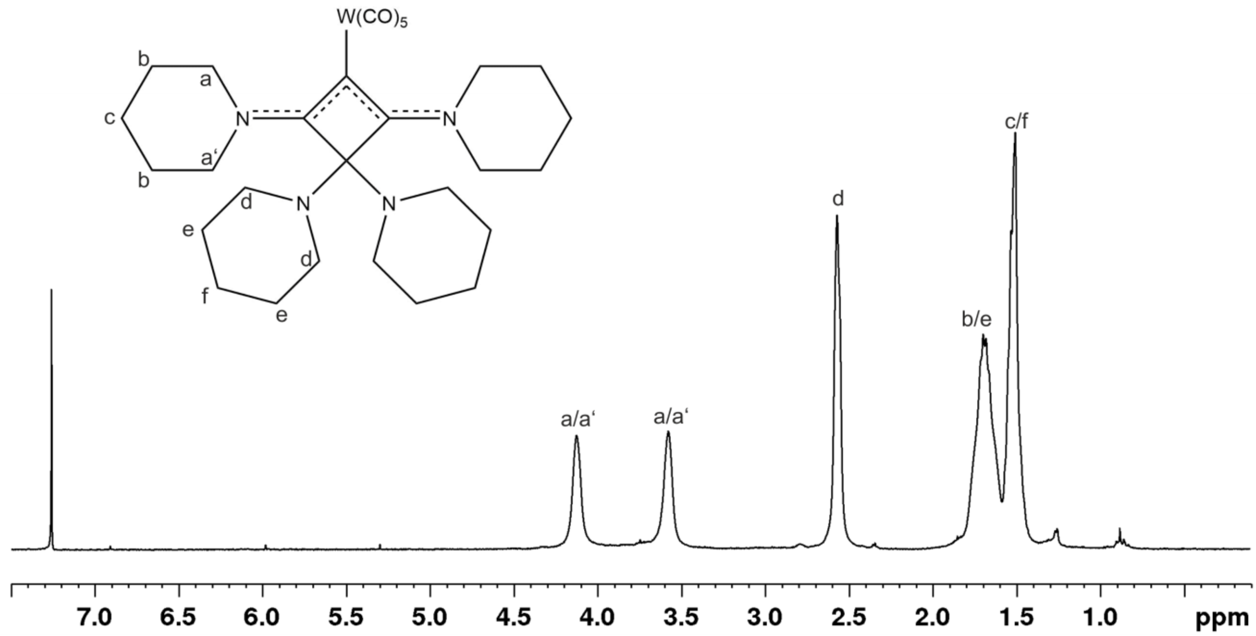

Fig. S28: ^{1}H-NMR ($CDCl_3$, 500 MHz, 300K) of **7**.

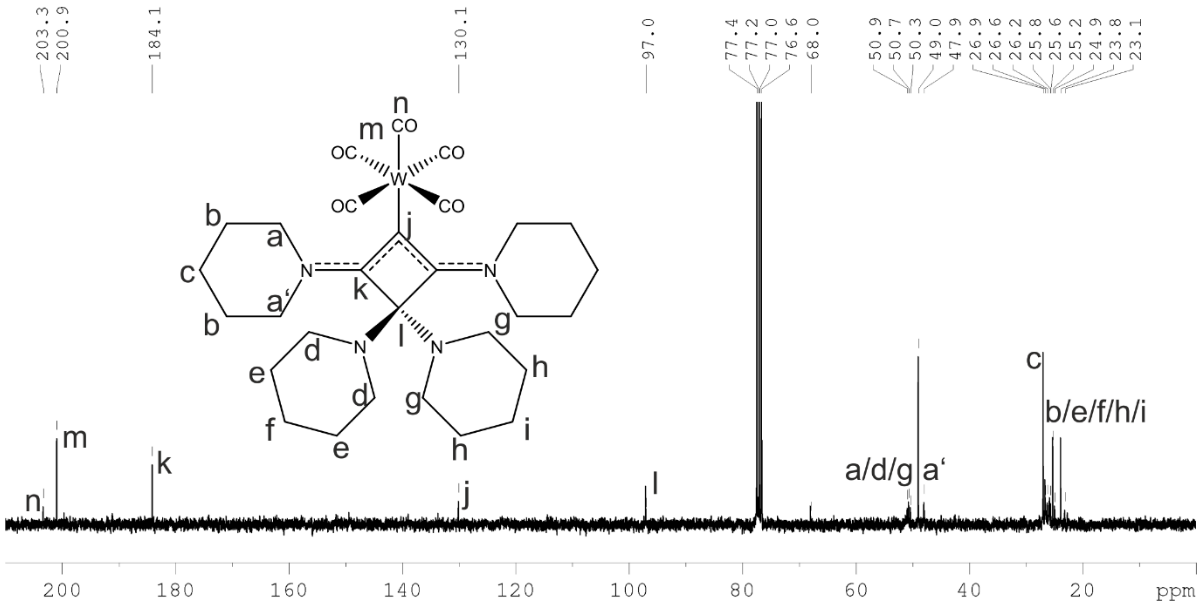

Fig. S29: ^{13}C-{^{1}H}-NMR ($CDCl_3$, 75.5 MHz, 300K) of **7**.

Buried Volume Calculations

The buried volume $\%V_{Bur}$ and also the topographic steric map for the CBA-ligand **5** were calculated from the solid state structure using the Web tool SambVCA2.[5] For the calculation the following settings were employed: Bondi radii scaled by 1.17, sphere radius = 3.5 Å, mesh spacing = 0.1. Au, Cl and the cocrystallised molecule of dichloromethane were deleted and H atoms excluded from the calculation.

The calculated values were compared to the literature values obtained for the calculation with a 2.0 Å M-C distance.

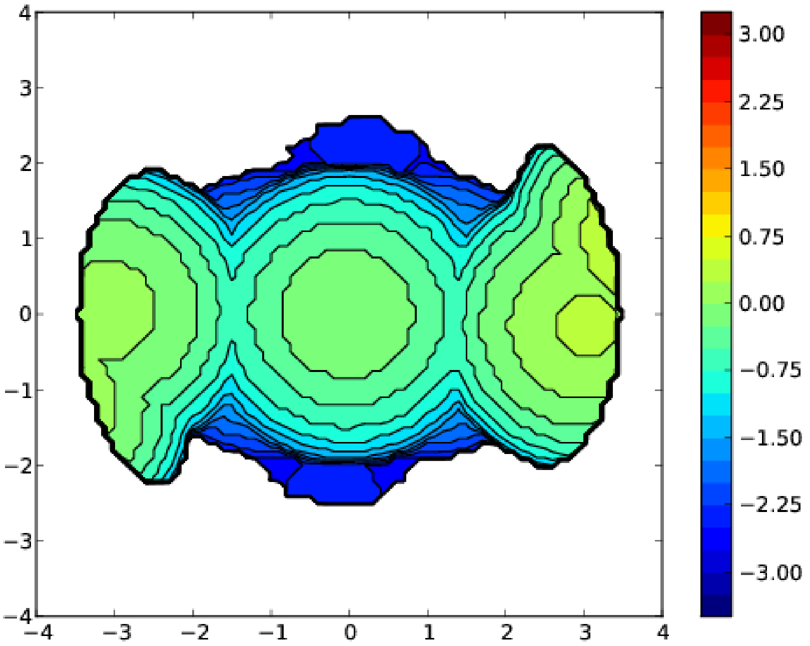

Fig. S30: Steric map calculated for the CBA-ligand in **5**.

Table S2: Results for the $\%V_{Bur}$ Calculations of the CBA-ligand in **5**

<table>
<tr><th colspan="3">V Free</th><th colspan="3">V Buried</th><th colspan="3">V Total</th><th colspan="3">V Exact</th></tr>
<tr><td colspan="3">124.7</td><td colspan="3">54.8</td><td colspan="3">179.5</td><td colspan="3">179.6</td></tr>
<tr><th colspan="4">%V_Free</th><th colspan="4">%V_Bur</th><th colspan="4">% Tot/Ex</th></tr>
<tr><td colspan="4">69.5</td><td colspan="4">30.5</td><td colspan="4">100.0</td></tr>
<tr><th colspan="2">Quadrant</th><th colspan="2">V_f</th><th colspan="2">V_b</th><th colspan="2">V_t</th><th colspan="2">%V_f</th><th colspan="2">%V_b</th></tr>
<tr><td colspan="2">SW</td><td colspan="2">31.2</td><td colspan="2">13.7</td><td colspan="2">44.9</td><td colspan="2">69.5</td><td colspan="2">30.5</td></tr>
<tr><td colspan="2">NW</td><td colspan="2">31.8</td><td colspan="2">13.1</td><td colspan="2">44.9</td><td colspan="2">70.9</td><td colspan="2">29.1</td></tr>
<tr><td colspan="2">NE</td><td colspan="2">30.7</td><td colspan="2">14.2</td><td colspan="2">44.8</td><td colspan="2">68.4</td><td colspan="2">31.6</td></tr>
<tr><td colspan="2">SE</td><td colspan="2">31.1</td><td colspan="2">13.8</td><td colspan="2">44.9</td><td colspan="2">69.3</td><td colspan="2">30.7</td></tr>
</table>

Computational Details

All computations to calculate the gas-phase electronic structures and energies for all species were performed using the density functional method B97-D (S. Grimme)[6] as implemented in the Gaussian09 program.[11] For all main group elements (C, H, N and Cl) the all-electron triple-ζ basis set 6-311G(d,p)[12] was used and for the main group metal tin the triple-ζ valence basis set plus polarization function Jorge-TZP[13] was applied.[14] The participating transition states (TS) are located at the same level of theory. Harmonic vibrational frequencies are calculated to characterize respective minima (reactants, intermediates, and products with no imaginary frequency) and first order saddle points (transition states with one imaginary frequency).

Table S3: Energies for all optimized structures

	Compound	$E_{0K}{}^{a}$ / [Ha]	$E_{298K}{}^{b}$ / [Ha]	$H_{298K}{}^{b}$ / [Ha]	$G_{298K}{}^{b}$ / [Ha]
educts	1,2-dipiperidino-acetylene (**1**)	-578.183049	-578.169062	-578.168118	-578.224484
	$SnCl_2$	-6944.204230	-6944.199968	-6944.199023	-6944.233706
dimerization of (**1**) to 3-buten-1-yne (**3**)	IN1	-1156.383929	-1156.354033	-1156.353089	-1156.448036
	TS1	-1156.366042	-1156.338055	-1156.337111	-1156.424702
	IN2	-1156.380415	-1156.352987	-1156.352042	-1156.438381
	TS2	-1156.379715	-1156.353265	-1156.352320	-1156.434891
	IN3	-1156.382103	-1156.355221	-1156.354277	-1156.438017
	1,3,4,4-pip_4-3-buten-1-yne (**3**)	-1156.444979	-1156.416766	-1156.415822	-1156.506890
formation of (**4a**)	IN1	-8100.731426	-8100.698663	-8100.697719	-8100.796121
	TS1	-8100.698408	-8100.665834	-8100.664889	-8100.763555
	IN2	-8100.696698	-8100.663198	-8100.662253	-8100.763972
	TS2	-8100.692294	-8100.659988	-8100.659044	-8100.756029
	(CBA)$SnCl_2$ (**4a**)	-8100.742988	-8100.710645	-8100.709701	-8100.807695
	(1,2,3,4-pip_4-1,3-cyclo-butadiene)$SnCl_2$ (**2a**)	-8100.753099	-8100.720471	-8100.719527	-8100.817237

[a] DFT energy incl. ZPE.
[b] standard conditions T = 298.15 K and p = 1 atm.

Molecular Orbitals of the free CBA-ligand

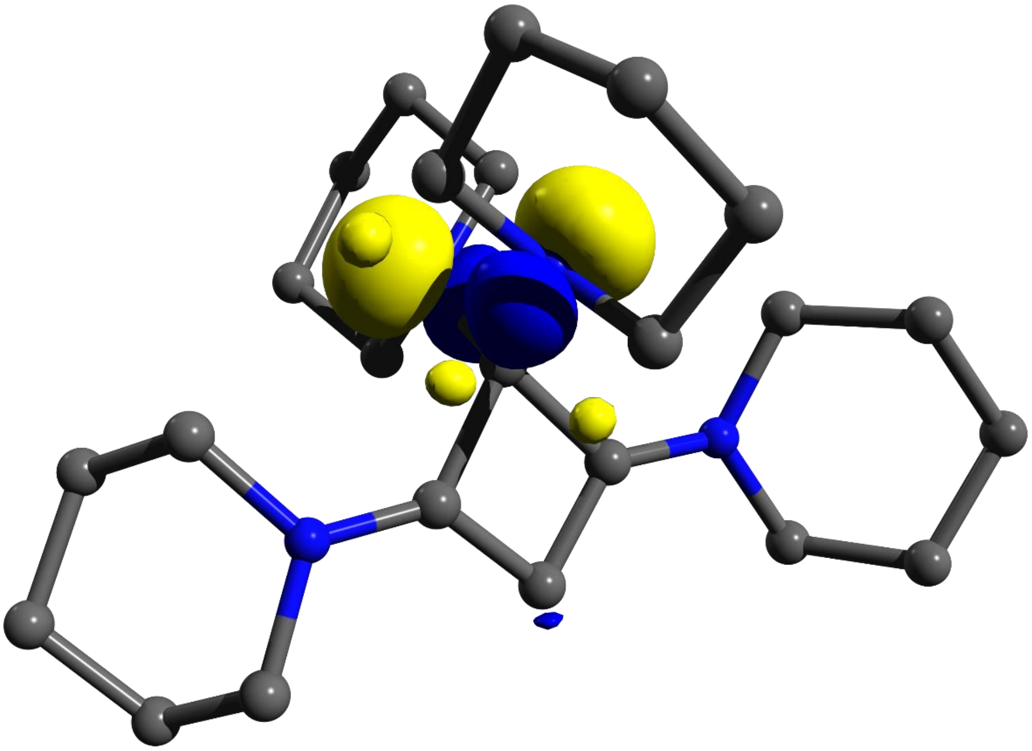

Fig. S31: Plot of the HOMO-2.

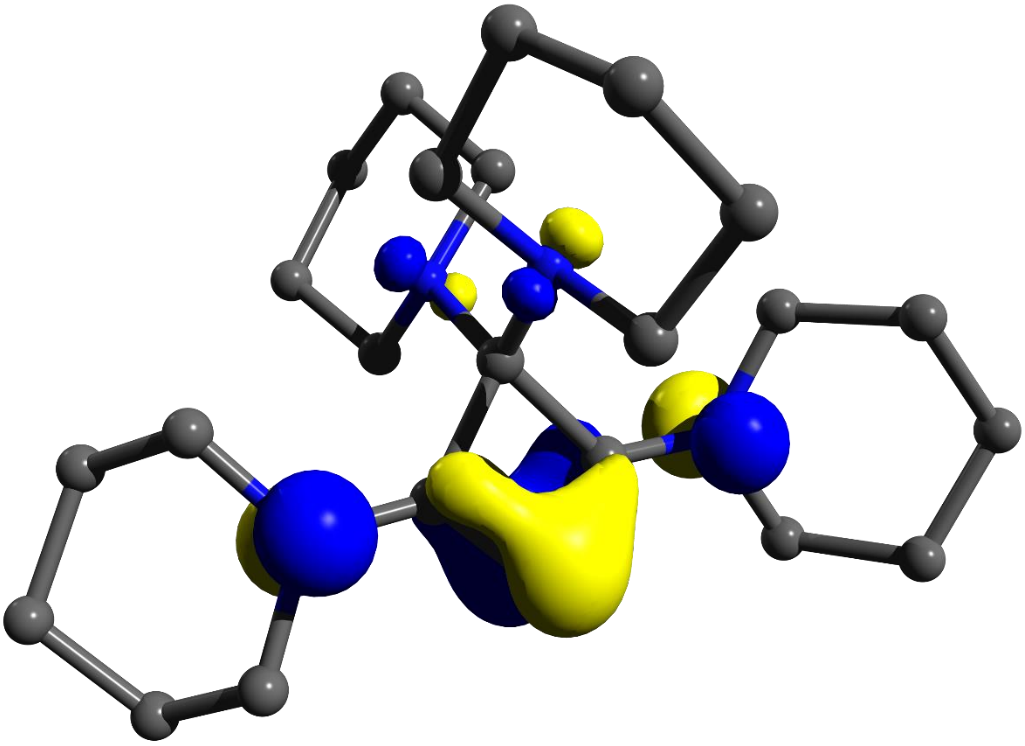

Fig. S32: Plot of the HOMO-1.

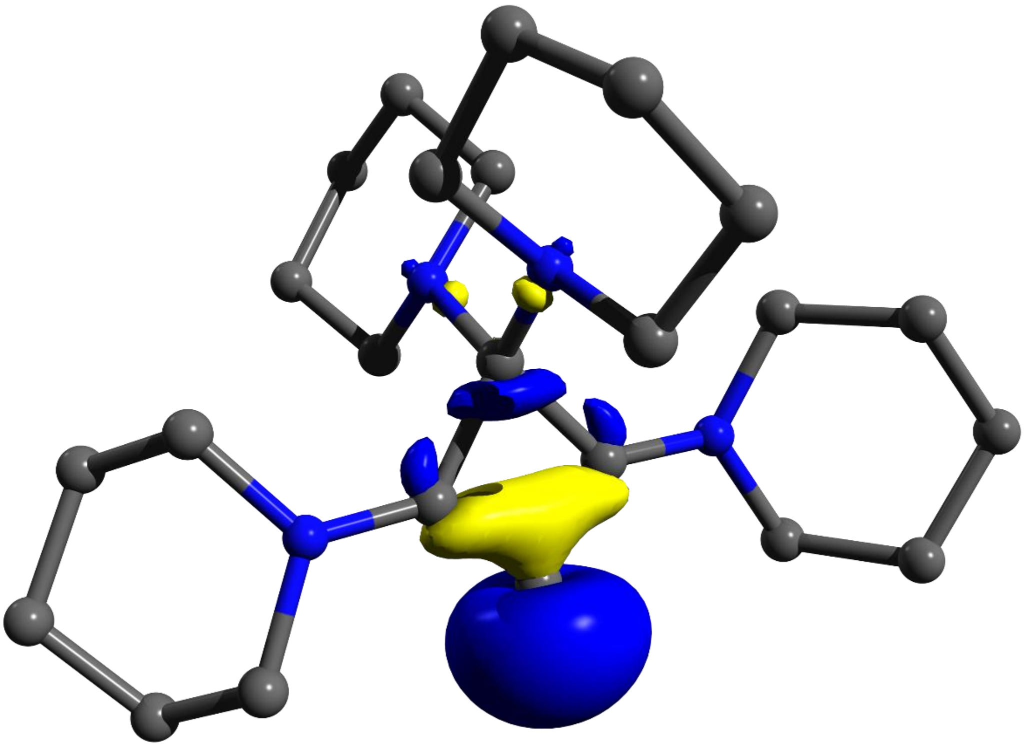

Fig. S33: Plot of the HOMO.

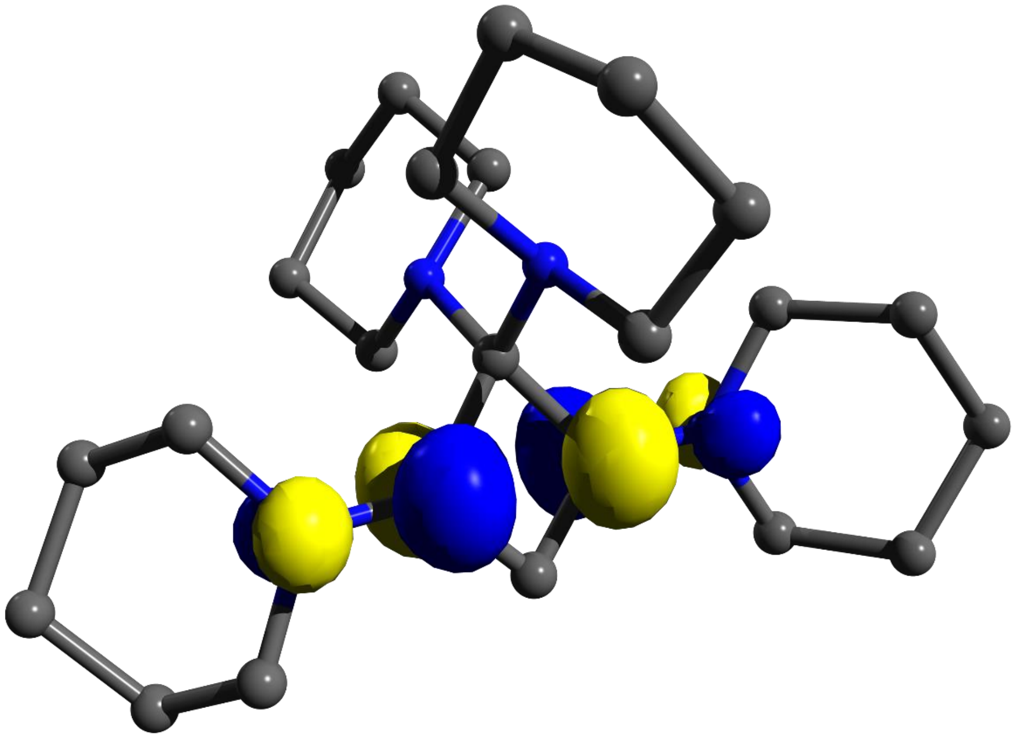

Fig. S34: Plot of the LUMO.

Energy Profiles

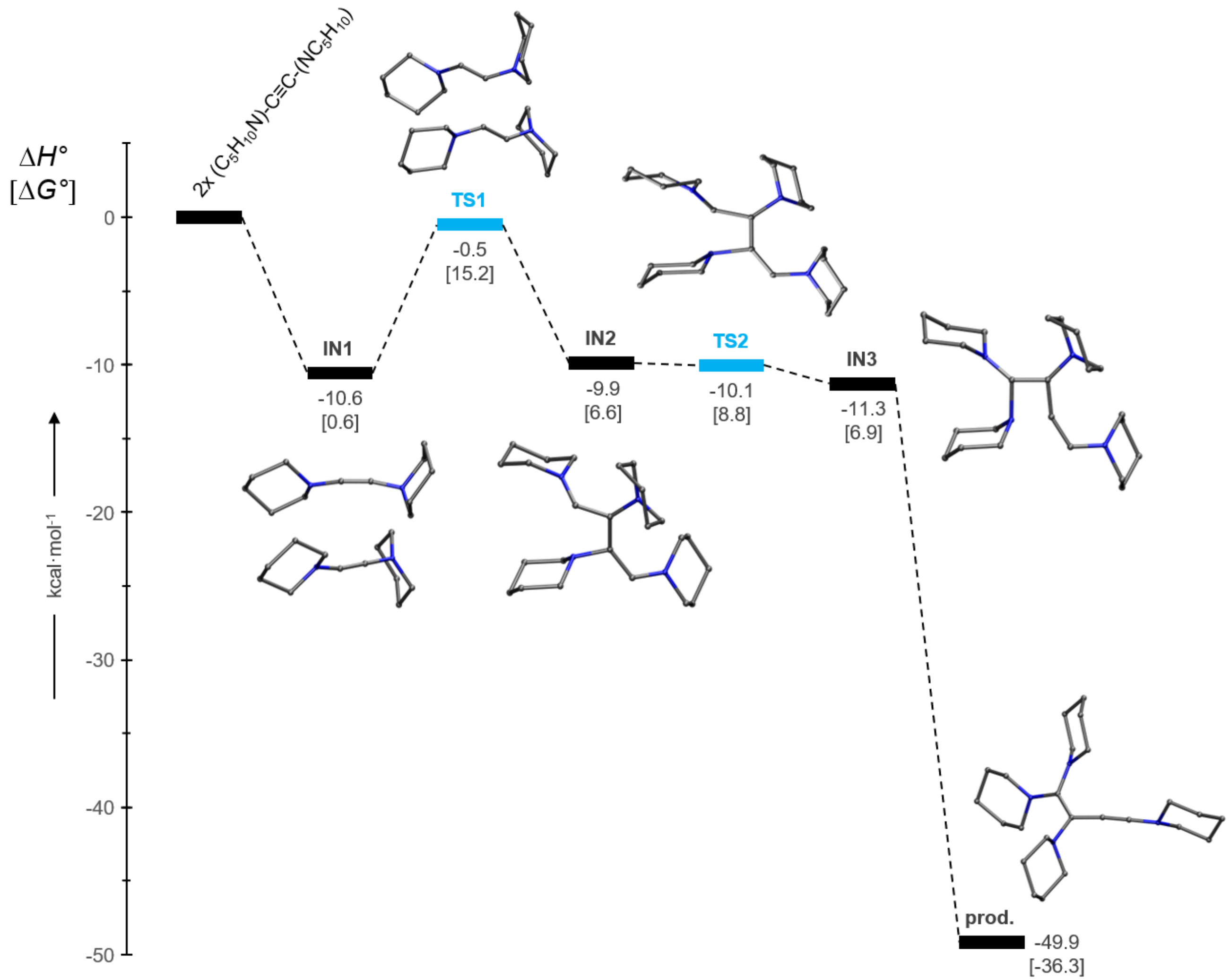

Fig. S35: Calculated energy profile (kcal mol^{-1}) of the dimerization of **1** to the enyne **3** at B97-D/6-311G(d,p) level.

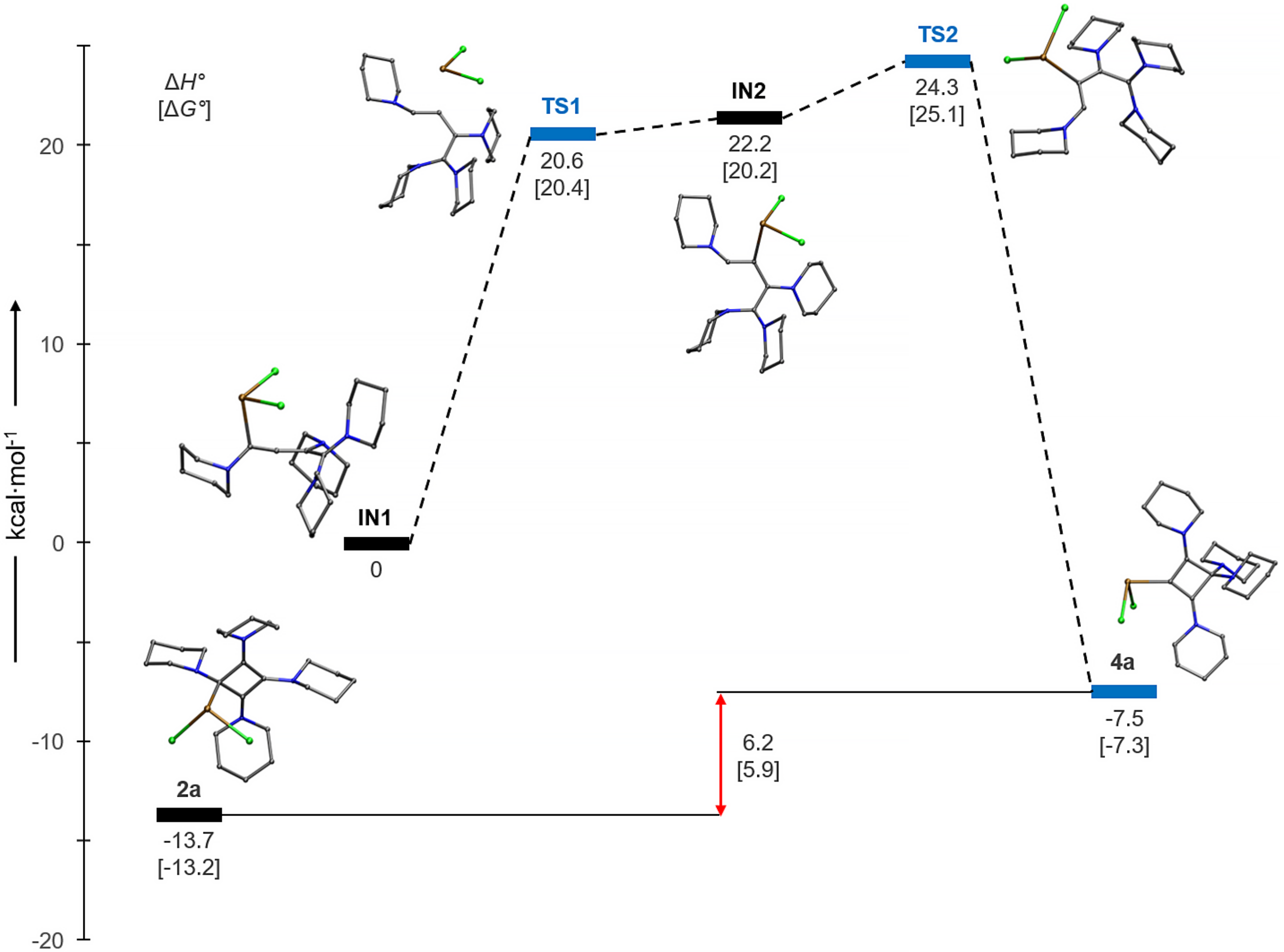

Fig. S36: Calculated energy profile (kcal mol^{-1}) of the Reaction of **3** with $SnCl_2$ at B97-D/6-311G(d,p) level with comparison of the energies of the isomeric compounds **2a** and **4a**.

Table S4. Selected experimental and calculated bond lengths and angles for the compounds **2a**, **3** and **4a**.

Bond length [Å]	**3**	**3**$_{calc}$	Bond length [Å]	**2a**	**2a**$_{calc}$	**4a**	**4a**$_{calc}$
C1-C2	1.193(2)	1.227	E–C1	2.320(3)	2.313	2.2444(2)	2.202
C2-C3	1.431(2)	1.417	C1–C2	1.558(5)	1.550	1.414(2)	1.406
C3-C4	1.369(2)	1.386	C1–C4	1.521(4)	1.520	1.421(2)	1.424
C3-N2	1.4486(14)	1.449	C2–C3	1.402(4)	1.412	1.555(2)	1.548
C4-N3	1.3949(13)	1.395	C3–C4	1.417(5)	1.426	1.568(2)	1.565
C4-N4	1.3944(14)	1.395	C4–N4	1.318(4)	1.313	1.320(2)	1.320
			C3–N3	1.402(5)	1.404	1.458(2)	1.460
Bond angle [°]			Bond angle [°]				
C2-C1-N1	176.6(3)	178.87	Cl1–E–Cl2	93.84(3)	99.89	93.310(17)	101.95
C2-C3-C4	123.08(10)	122.54	C1–E–Cl1	93.08(8)	92.78	94.19(5)	94.10
N2-C3-C2	118.31(9)	119.42	C1–E–Cl2	96.88(9)	96.32	94.62(5)	89.01
N3-C4-N4	112.69(9)	113.15	C2–C1–C4	81.3(2)	82.04	89.86(19)	90.01

[1] A. R. Petrov, C. G. Daniliuc, P. G. Jones, M. Tamm, *Chem. Eur. J.* **2010**, *16*, 11804.
[2] W. Strohmeier, J. F. Guttenberger, H. Blumenthal, G. Albert, *Chem. Ber.* **1966**, *99*, 3419.
[3] R. Uson, A. Laguna, M. Laguna, *Inorg. Synth.* **1989**, 86.
[4] a) G. M. Sheldrick, *Acta crystallographica. Section A, Foundations of crystallography* **2008**, *64*, 112; b) G. M. Sheldrick, *Acta crystallographica. Section C, Structural chemistry* **2015**, *71*, 3.
[5] L. Falivene, R. Credendino, A. Poater, A. Petta, L. Serra, R. Oliva, V. Scarano, L. Cavallo, *Organometallics* **2016**, *35*, 2286.
[6] J. Antony, S. Grimme, *Phys. Chem. Chem. Phys.* **2006**, *8*, 5287.
11 Gaussian 09, Revision D.01, M. *J*. Frisch, G. W. Trucks, H. B. Schlegel, G. E. Scuseria, M. A. Robb, *J*. R. Cheeseman, G. Scalmani, V. Barone, G. A. Petersson, H. Nakatsu*J*i, X. Li, M. Caricato, A. Marenich, *J*. Bloino, B. G. *J*anesko, R. Gomperts, B. Mennucci, H. P. Hratchian, *J*. V. Ortiz, A. F. Izmaylov, *J*. L. Sonnenberg, D. Williams-Young, F. Ding, F. Lipparini, F. Egidi, *J*. Goings, B. Peng, A. Petrone, T. Henderson, D. Ranasinghe, V. G. Zakrzewski, *J*. Gao, N. Rega, G. Zheng, W. Liang, M. Hada, M. Ehara, K. Toyota, R. Fukuda, *J*. Hasegawa, M. Ishida, T. Naka*J*ima, Y. Honda, O. Kitao, H. Nakai, T. Vreven, K. Throssell, *J*. A. Montgomery, *J*r., *J*. E. Peralta, F. Ogliaro, M. Bearpark, *J*. *J*. Heyd, E. Brothers, K. N. Kudin, V. N. Staroverov, T. Keith, R. Kobayashi, *J*. Normand, K. Raghavachari, A. Rendell, *J*. C. Burant, S. S. Iyengar, *J*. Tomasi, M. Cossi, *J*. M. Millam, M. Klene, C. Adamo, R. Cammi, *J*. W. Ochterski, R. L. Martin, K. Morokuma, O. Farkas, *J*. B. Foresman, and D. *J*. Fox, Gaussian, Inc., Wallingford CT, **2016**.
12 R. Krishnan, J. S. Binkley, R. Seeger and J. A. People, *J. Chem. Phys.* **1980**, *72*, 650.
13 C. T. Campos and F. E. Jorge, *Mol. Phys.* **2013**, *111*, 167.
14 Jorge-TZP basis sets (for Sn) was obtained from the Extensible Computational Chemistry Environment Basis Set Database, Version 1.2.2 [https://bse.pnl.gov/bse/portal]. a) The Role of Databases in Support of Computational Chemistry Calculations, D. Feller, *J. Comp. Chem.* **1996**, *17*, 1571. b) Basis Set Exchange: A Community Database for Computational Sciences, K. L. Schuchardt, B. T. Didier, T. Elsethagen, L. Sun, V. Gurumoorthi, J. Chase, J. Li and T. L. Windus, *J. Chem. Inf. Model.* **2007**, *47*, 1045.
